# Recent advances in understanding the immune microenvironment in ovarian cancer

**DOI:** 10.3389/fimmu.2024.1412328

**Published:** 2024-06-05

**Authors:** Jinxin Chen, Lu Yang, Yiming Ma, Ye Zhang

**Affiliations:** ^1^ Department of Gynecology, Cancer Hospital of Dalian University of Technology, Cancer Hospital of China Medical University, Liaoning Cancer Hospital & Institute, Shenyang, China; ^2^ Department of Internal Medicine, Cancer Hospital of Dalian University of Technology, Cancer Hospital of China Medical University, Liaoning Cancer Hospital & Institute, Shenyang, China; ^3^ Department of Medical Oncology, Liaoning Cancer Hospital & Institute, Shenyang, Liaoning, China; ^4^ Liaoning Key Laboratory of Gastrointestinal Cancer Translational Research, Shenyang, Liaoning, China; ^5^ Department of Radiation Oncology, Cancer Hospital of Dalian University of Technology, Cancer Hospital of China Medical University, Liaoning Cancer Hospital & Institute, Shenyang, China

**Keywords:** ovarian cancer, tumor environment, immune cells, immune checkpoint inhibitor, immunotherapy

## Abstract

The occurrence of ovarian cancer (OC) is a major factor in women’s mortality rates. Despite progress in medical treatments, like new drugs targeting homologous recombination deficiency, survival rates for OC patients are still not ideal. The tumor microenvironment (TME) includes cancer cells, fibroblasts linked to cancer (CAFs), immune-inflammatory cells, and the substances these cells secrete, along with non-cellular components in the extracellular matrix (ECM). First, the TME mainly plays a role in inhibiting tumor growth and protecting normal cell survival. As tumors progress, the TME gradually becomes a place to promote tumor cell progression. Immune cells in the TME have attracted much attention as targets for immunotherapy. Immune checkpoint inhibitor (ICI) therapy has the potential to regulate the TME, suppressing factors that facilitate tumor advancement, reactivating immune cells, managing tumor growth, and extending the survival of patients with advanced cancer. This review presents an outline of current studies on the distinct cellular elements within the OC TME, detailing their main functions and possible signaling pathways. Additionally, we examine immunotherapy rechallenge in OC, with a specific emphasis on the biological reasons behind resistance to ICIs.

## 1 Introduction

Ovarian cancer (OC) is a prevalent form of gynecological cancer, recognized for its aggressive nature and tendency to metastasize (1). Its atypical presentation poses challenges for early detection and treatment, with approximately three-quarters of OC cases diagnosed at an advanced stage (2). OC includes various types, including epithelial tumors, sex cord stromal tumors, germ cell tumors, unclassified types, and metastatic secondary tumors, with epithelial ovarian cancer (EOC) accounting for over 95% of cases (3). EOC comprises diverse histological types, grades, and molecular profiles, primarily classified into type I and type II. Type I OC typically includes endometrioid carcinoma, low-grade serous carcinoma, clear cell carcinoma, and mucinous carcinoma, often arising from atypical proliferative (borderline) tumors (4). Type I OC is associated with mutations in genes such as K-Ras and PTEN, tends to present at early stages, exhibits slow growth, and carries a favorable prognosis. Conversely, type II OC originates from serous intraepithelial carcinoma of the fallopian tube, featuring high-grade serous carcinoma (HGSC), carcinosarcoma, and undifferentiated carcinoma subtypes (5). Research indicates that inflammation and endometriosis stemming from repeated ovarian cycles contribute to the development of type II OC. This type is frequently linked to mutations in the p53 and BRCA genes, HER2 overexpression, and is often diagnosed late, leading to a poor prognosis. It tends to be highly invasive and carries a high mortality rate (6). Currently, the primary approach to treating OC involves surgical resection alongside systemic radiotherapy and chemotherapy (7). In recent years, increased understanding of tumor immunity has led to the recognition of immunotherapy as a promising therapeutic option (8, 9). Despite the effectiveness of many treatments in managing OC, the disease still exhibits high rates of recurrence and low survival rates, underscoring the need for the development of new or enhanced therapeutic strategies.Tumor development is a complex process that unfolds in multiple stages. The tumor microenvironment (TME) plays a crucial role in facilitating the uncontrolled survival and growth of tumor cells, from initial carcinogenesis to fully developed cancer. Comprising various cell types and their driver molecules, such as immune cells, interstitial cells, endothelial cells, adipocytes, extracellular matrix, cytokines, and chemokines, the TME orchestrates diverse intracellular signaling pathways (10). Recent investigations underscore the significance of both the primary site TME and the microenvironment formed by distant metastasis in driving tumor proliferation, metastasis, invasion, drug resistance, and the preservation of tumor cell stemness (11). Immune checkpoints (ICs) are molecules expressed on immune cells that modulate immune system activation. Immune checkpoint inhibitors (ICIs) act by blocking the interaction between ICs and their ligands; thus, preventing T lymphocyte inactivation and exerting an anti-tumor effect.

In the past two decades, targeting the TME has become a key therapeutic strategy for solid tumors (12). The first successful pathway involved the programmed death 1 (PD-1)/programmed death ligand 1 (PD-L1) and cytotoxic T lymphocyte-associated protein 4 (CTLA-4) pathways, which are key mediators through which cancer cells evade antitumor T-cell-mediated cytotoxicity (13). Immune checkpoint inhibitors (ICIs) targeting PD-1/PD-L1 and CTLA-4 were the earliest developed, showing remarkable benefits in specific patients and revolutionizing the treatment landscape for numerous cancers (14). While these ICIs were also trialed in OC patients, phase III trials demonstrated that ICIs, either as monotherapy or combined with chemotherapy, did not yield statistically significant survival advantages (15, 16). The strong immunosuppressive environment and the number of participants involved in the OC TME are likely reasons for these disappointing results. Current data suggests that targeting the OC TME remains a challenging endeavor. However, within the context of the OC TME, concentrating solely on T cell activity and the PD-1/PD-L1 pathway might be overly restrictive. Achieving a broader characterization and a more thorough comprehension of the intricate interactions between OC tumor cells and their microenvironment is imperative to altering this trend.

This review summarized the related studies of different cell populations that combined the TME and immunotherapy challenge in OC.

## 2 Overview of the TME

**Figure 1:**
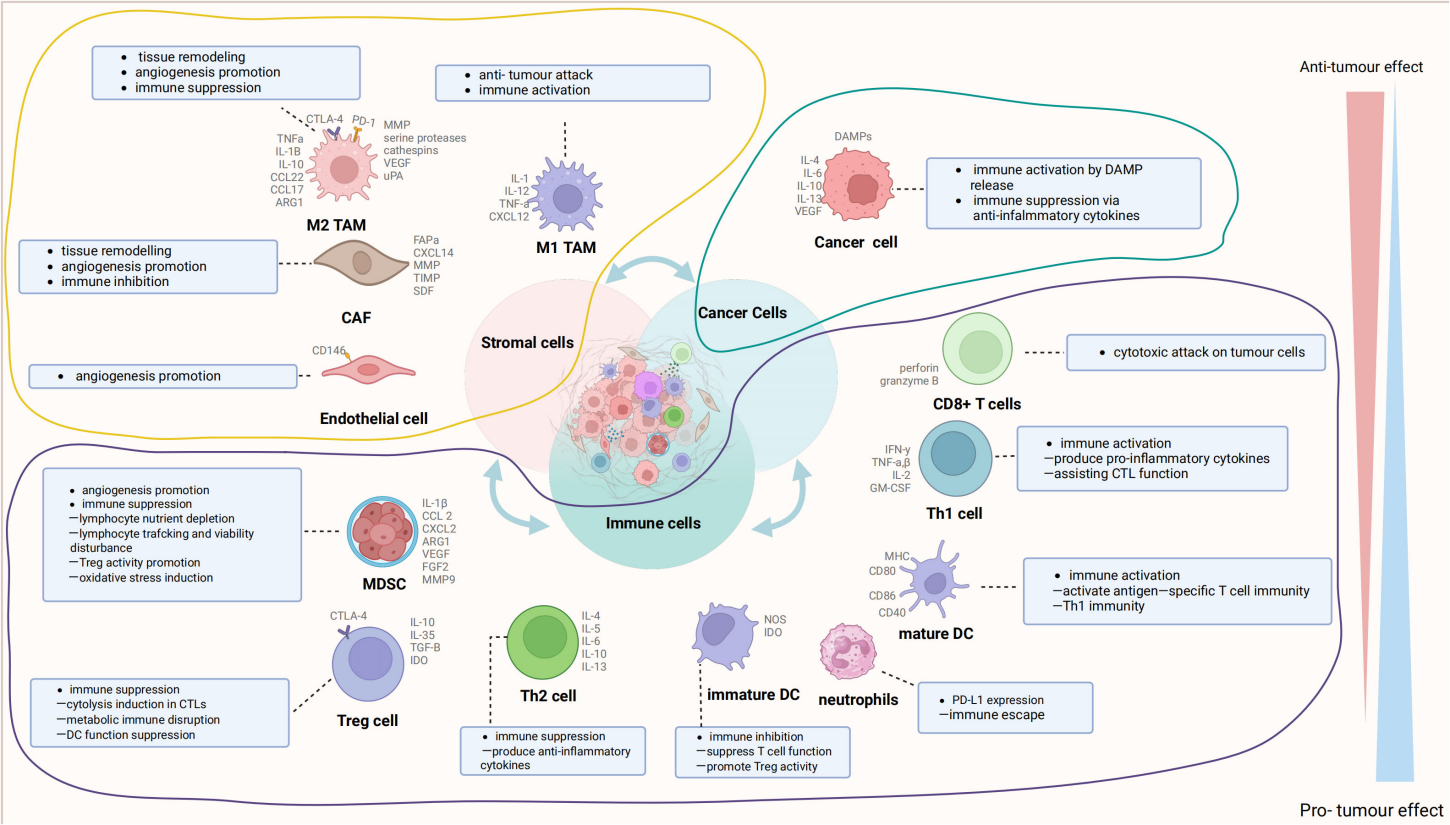
The cell com ponents in the tumor microenvironment (TME). The TME is mainly composed of cancer cells, immune cells, and stromal cells. These cells interact with receptors such as the molecules they secrete, histocompatibility complex (MHC) molecules, programmed cell death protein-1 (PD-1), and others to form an evolutionary microenvironment. The figure was generated by Biorender.com. TAMs, tumor-associated macrophages; DCs, dendritic cells; MDSCs, myeloid-derived suppressor cells; CAFs, cancer-associated fibroblasts.

The TME is the cellular milieu in which neoplastic cells or cancer stem cells reside. These include the blood vessels surrounding tumor cells, immune cells, other nontumor cells, the extracellular matrix, and signaling molecules (17, 18). The interactions between these components and tumor cells have a significant impact on tumor progression (19, 20). The TME consists of cancer cells, the extracellular matrix (ECM), cancer-associated fibroblasts (CAFs), a complex network of blood vessels, and diverse immune cells, including T-cells, B-cells, and cells linked to tumor progression (TACs). Cancer cells recruit and activate immune cells and stromal components, like lymphocytes, tumor-associated macrophages (TAMs), natural killer (NK) cells, dendritic cells (DCs), tumor-associated neutrophils (TANs), and myeloid-derived suppressor cells (MDSCs) (21–23), collectively establishing an anti-tumor inflammatory microenvironment during early tumor colonization or expansion; thus, impeding tumor growth (24, 25). However, prolonged exposure to tumor antigens and immune activation can deplete or alter effector cells, resulting in an immunosuppressive microenvironment that fosters tumor aggressiveness (26, 27). Given that the key cellular components sustaining this immunosuppressive microenvironment also exhibit anti-cancer properties during the initial tumor phases, they represent potential intervention targets. **Figure 1** illustrates the cellular constituents within the TME.

CAFs are significant components of the tumor stroma and are primarily distributed in the perivascular or tumor peripheral fibrous mesenchyme (28, 29). CAFs produce cytokines, ECM components, and associated enzymes (30, 31). The ECM offers structural support to TME cells and plays a crucial role in cellular adhesion and infiltration (32, 33). ECM deposition leads to the creation of a dense fibrous mesenchyme enveloping the tumor, making the tumor tissue stiffer and more brittle than normal tissue. The buildup of ECM results in the formation of a dense fibrous mesenchyme encircling the tumor, increasing its stiffness and fragility compared to normal tissue. This forms a physical barrier hindering immune cell penetration and obstructing the efficient delivery of anti-cancer drugs to the TME. CAFs produce matrix metalloproteinases that can modify the ECM and release chemokines, growth factors, and proangiogenic factors, thereby facilitating the malignant transformation of tumors (34–36). Due to the rapid growth of tumors and irregular blood flow patterns, tumors often face inadequate blood supply, leading to prolonged oxygen deprivation. Consequently, the tumor environment becomes acidic due to metabolic processes generating lactate and hydrogen ions. Vascular abnormalities and metabolic imbalances trigger signaling cascades, fostering the development of an immunosuppressive TME. This environment is infiltrated by diverse immune cells, including CD8+ or cytotoxic T lymphocytes (CTLs), crucial for tumor elimination. While CD8+ or cytotoxic T lymphocytes (CTLs) target tumor cells for destruction, regulatory T cells (Tregs) suppress the activity of effector T cells and promote immunosuppression within the TME (37–39). M1-type macrophages typically release Th1 cytokines, exerting pro-inflammatory and anti-tumor effects (40, 41). However, TAMs in the TME predominantly exhibit the M2 subtype and can induce angiogenesis and tumor invasion by secreting Th2 cytokines (42, 43). NK cells can eliminate target cells through the release of granzymes and perforin or by facilitating antibody-dependent cytotoxicity via their Fc receptors (44, 45). Nevertheless, the killing activity of T cells is hindered by the accumulation of TGF-β in the TME, and the antigen-presenting function of DCs is impaired by the hypoxic and inflammatory conditions of the TME (46, 47). Additionally, MDSCs, acting as negative immune regulators within the TME, suppress T-cell activation and the functions of various immune cells (48, 49).

## 3 The characteristics of the TME in OC

The participation of the TME is pivotal in the advancement and dissemination of OC. Substances released within the OC TME interact with tumor cells, facilitating their invasion and metastasis (50). Moreover, the TME holds potential as both a diagnostic biomarker and a target for therapeutic intervention in OC (51). Research has indicated that CAFs stimulate the elevation of pyruvate dehydrogenase kinase 1 (PDK1) expression in cancer cells through proteins secreted within the TME. PDK1 regulates metabolism and enhances cellular adhesion, migration, invasion, angiogenesis, and anchorage-independent growth of OC cells. This, in turn, leads to tumor invasion and migration (52). TAMs often infiltrate ascites from patients with advanced OC. The overexpression of TAMs has been linked to a negative prognosis for patients with tumors. Research findings suggest that TAMs boost tumor angiogenesis by secreting factors such as vascular endothelial growth factor (VEGF), tumor growth factor-β (TGF-β), matrix metalloproteinases, hypoxia-inducible factors, and adrenomedullin (53). TAMs also release growth factors that facilitate the growth and early metastasis of OC (54). Additionally, TAMs contribute to an immunosuppressive microenvironment by releasing modulators that affect T-cells, thereby aiding tumor immune evasion (55). The interplay between the TME and tumor cells regulates the initiation, progression, and metastasis of OC. Some TME factors can influence how OC patients respond to treatment. Studies have shown that altering the TME can improve the efficacy of OC chemotherapy (56, 57). However, immune effector cells within the TME face inhibition not only from tumor cells but also from regulatory T cells (Tregs), immature DCs, MDSCs, and TAMs. This fosters an immunosuppressive microenvironment conducive to immune evasion (58, 59). The TIME can impact the development of OC cells. Both immune and targeted therapies against the TIME have shown promising results. Additionally, reversing the suppressive immune microenvironment in combination with antiangiogenic therapeutic regimens holds promise as a potential treatment for recurrent ovarian epithelial cancers (60, 61). The TIME could influence the development and progression of OC, which can ultimately affect patient survival.

## 4 The biological roles and functions of different immune cells in OC

### 4.1 TAMs

TAMs constitute a specific subset of macrophages crucial for shaping the TME. TAMs are commonly present in various tumor types and exhibit pro-tumorigenic activities, significantly influencing angiogenesis, metastasis, drug resistance, and anti-tumor immune suppression (62). Macrophages have the ability to polarize into two distinct activation states known as classical activation (M1 phenotype) and alternative activation (M2 phenotype) (63–65). M1 TAMs, also known as inflammatory macrophages, are stimulated by factors like IFN-γ, TNF-α, and lipopolysaccharide (LPS). They secrete pro-inflammatory cytokines such as TNF-α, IL-1β, IL-12, IL-18, and nitric oxide, exerting phagocytic and cytotoxic effects on target cells (66). Additionally, M1-like TAMs exhibit increased expression of MHC-II, CD68, CD80, and CD86 co-stimulatory markers. Conversely, M2-like TAMs, also known as macrophages with anti-inflammatory properties, are primarily induced by cytokines like IL-4, IL-13, CSF-1, IL-10, and TGF-β. M2-type TAMs produce anti-inflammatory mediators such as IL-10 and TGF-β, participate in type II immune responses, and promote tumor angiogenesis, proliferation, invasion, and metastasis. Phenotypically, M2-type TAMs express surface markers like CD206, CD163, and Arg1 (67). Parayatha et al. demonstrated the specific localization of TAMs within the peritoneal cavity of mice bearing homozygous ID8-VEGF ovarian carcinoma using nanoparticles composed of hyaluronic acid and encapsulating miR-125b (HA-PEI-miR-125b). This resulted in the repolarization of macrophages towards an immune-activated phenotype. Combining intraperitoneal injection of paclitaxel with HA-PEI-miR-125b nanoparticles augmented the anti-tumor efficacy of paclitaxel during late-stage disease progression. The study findings suggest that HA-PEI-miR-125b nanoparticles are well-tolerated and warrant further investigation in clinical trials. Bai et al. (68) observed a direct correlation between the expression level of CTHRC1 and the degree of endosomal infiltration by M2-like CD68+ CD163+ TAMs, accompanied by increased STAT6 phosphorylation in EOC. Furthermore, recombinant CTHRC1 protein (rCTHRC1) induced a dose-dependent M2-like macrophage phenotype, as evidenced by STAT6 signaling pathway activation. The conditioned cultures of Lenti-CTHRC1 EOC cells promoted macrophage M2 polarization, while CTHRC1 knockdown eliminated STAT6-mediated macrophage M2 polarization. This study suggested that CTHRC1 may have a significant impact on regulating macrophage M2 polarization in the ovarian TME, indicating its potential as a therapeutic target for antitumor immunity (69). Xia et al. revealed that Tim-4+ TAMs originate from embryonic sources and persist locally. They also observed that these Tim-4+ TAMs promote tumor growth *in vivo* and display increased oxidative phosphorylation. Additionally, Tim-4+ TAMs adapt to counter oxidative stress through mitosis. Depletion of Tim-4+ TAMs via ROS-induced apoptosis, resulting from genetic defects in the 200 kDa autophagy-related FAK family interacting protein, enhances T-cell immunity and suppresses ID8 tumor growth *in vivo*. Furthermore, the study noted similarities between human ovarian cancer-associated macrophages expressing complement receptor (CRIg) and murine TAMs expressing Tim-4 in terms of transcriptional profile, metabolism, and function. These findings suggest that targeting CRIg-positive (Tim-4-positive) TAMs could be a promising therapeutic approach for ovarian cancer patients with peritoneal metastases (70). Hoover et al. developed the IKFM transgenic mouse model to assess the impact of increased macrophage NF-κB activity on synthetic mouse models of TBR5 and ID8-Luc OC during two distinct timeframes: 1) established tumors; and 2) during tumor implantation and early tumor growth. Upon sacrifice, various parameters, including tumor weight, ascites volume, ascites supernatant and cells, and solid tumors were collected. Immunofluorescence staining and qPCR analyses were employed to investigate macrophage and T-cell populations in solid tumors and/or ascites. Additionally, ELISA was used to analyze soluble factors in ascites. Comparisons between the control and IKFM groups were performed using the two-tailed Mann−Whitney test (71). Ardighieri et al. discovered that immunoconverted HGSCs contain CXCL10-producing M1-type TAMs that closely resemble T cells. A subset of these M1-type TAMs also coexpressed TREM2. M1-polarized TAMs were almost undetectable in T-cell-poor clear cell carcinoma (CCC). Single-cell RNA sequencing confirmed the overexpression of antigen processing and gene expression programs by CXCL10+ IRF1+ STAT1+ M1-type TAMs within tumors. These results support the clinical significance of the CXCL10+ IRF1+ STAT1+ macrophage subset as a potential biomarker indicating the activation of T cells within tumors. This discovery offers a novel approach to identify patients who may have a greater likelihood of responding positively to immunotherapy targeting T cells or macrophages. In a study conducted by Le et al. (72), M2 macrophage supernatant was shown to have a modest enhancing effect on the proliferation, invasion, and migration abilities of A2780/DDP cells. However, this effect was counteracted in a manner that varied with the dose of TPL administered. Notably, when TPL was combined with cisplatin (DDP), TPL significantly reduced the tumor burden and prolonged survival in mice through its ability to inhibit the polarization of M2 macrophages and downregulate the expression of CD31 and CD206. Additionally, the sequencing results revealed that DDP upregulated Akkermansia, while TPL upregulated Clostridium. Notably, the combined effect of DDP and TPL led to the decrease in Lactobacillus and Akkermansia abundance. These findings underscore the importance of M2 TAMs in the migratory capacity, invasiveness, and tolerance to DDP in EOC, as well as elucidate the mechanism by which TPL reverses M2 macrophage polarization (73). Long et al. uncovered a direct correlation between the malignancy level of ovarian cancer (OvCa) cells and the formation of OvCa-TAMs spheroids. They also identified that CCL18 induces macrophage colony-stimulating factor (M-CSF) transcription via zinc E box binding isozyme 1 (ZEB1) in OvCa cells, subsequently driving the polarization of M2-TAMs. Thus, a reciprocal interaction loop involving CCL18-ZEB1-M-CSF was elucidated between OvCa cells and TAMs within spheroids. The study proposes that the formation of OvCa-TAMs spheroids leads to an invasive phenotype of OvCa cells, constituting one of the specific feedback loops of CCL18-ZEB1-M-CSF. Inhibition of ZEB1 reduces OvCa-TAMs spheroids in ascites, impedes OC metastasis, and enhances the prognosis of OC patients (74). Wu et al. demonstrated that the administration of BETi resulted in a significant increase in programmed cell death among THP-1 monocytes and macrophages. Moreover, BETi selectively hindered the viability of CCR2+ macrophages while inducing their transition into a phenotype resembling M1 cells. RNA-seq analysis unveiled that BETi specifically targeted cytokines and chemokines associated with macrophage inflammation in ovarian cancer. The combined application of ABBV-075 (a BET inhibitor) and bevacizumab demonstrated enhanced efficacy in suppressing tumor growth, reducing infiltration by macrophages, and prolonging the lifespan of mice bearing tumors compared to that of the control groups or individual treatments. This study suggested that BETi plays some role in selectively targeting CCR2+ TAMs and improving the effectiveness of AVA in treating OC. Khan et al. uncovered that VSSP reduced peritoneal TAMs and prompted M1-like polarization of TAMs in an ID8 systemic model of EOC. Moreover, VSSP treatment mitigated the suppressive effects of peritoneal TAMs and granulocytes on CD8+ T cell responses to ex vivo stimuli. Additionally, ex vivo exposure to VSSP induced M1-like polarization of TAMs derived from patients with metastatic OC and differentially alleviated their suppressive phenotype. These findings indicate that VSSP reshapes myeloid responses, disrupting the inhibitory pathway and potentially enhancing the efficacy of VSSP administration in the TME to improve anti-tumor immunity (75). Werehene et al. found that increased expression of epithelial pGSN was linked to the apoptosis of M1 macrophages via augmented activation of caspase-3 and reduced production of iNOS and TNFα. Furthermore, independent prognostic analysis demonstrated that epithelial pGSN expression could forecast progression-free survival. These results suggest that pGSN regulates inflammation by modulating the abundance and function of various macrophage subtypes within the ovarian TME. Thus, targeting pGSN may offer a promising therapeutic avenue to overcome immune-mediated chemotherapy resistance in OVCA (76). Li et al. unveiled that a novel circular RNA, circITGB6, exhibited significant elevation in tumor tissues and sera of platinum-resistant OC patients. Mechanistic investigations revealed that circITGB6 directly interacts with IGF2BP2 and FGF9 mRNA, forming a circITGB6/IGF2BP2/FGF9 RNA-protein ternary complex in the cytoplasm. This complex stabilizes FGF9 mRNA and induces the polarization of TAMs toward the M2 phenotype. Furthermore, *in vivo* reversal of OC CDDP resistance was observed upon blocking circITGB6-induced M2 macrophage polarization using antisense oligonucleotides targeting circITGB6. These findings unveil a novel mechanism of platinum resistance in OC and suggest that circITGB6 could serve as a promising prognostic marker and therapeutic target for patients with this disease (62). Chen et al. revealed that myricetin inhibits the alternatively activated (M2) polarization of TAMs and reduces the secretion of tumorigenic factors by TAMs, thereby counteracting the pro-tumorigenic effect of TAMs on OC cells. Furthermore, cardamonin inhibited tumor growth and decreased the expression of CD163 and CD206 in xenografted nude mice. Additionally, STAT3 was found to be closely associated with mTOR activity. To conclude, these findings suggest that myricetin has the potential to inhibit the protumor function of TAMs by reducing M2 polarization through the inhibition of mTOR. Therefore, OC patients could benefit from the use of myricetin as a promising therapeutic intervention. Chen (77) et al. conducted a bioinformatics analysis to screen for DEGs associated with OC. They identified NEAT1 as a highly expressed gene in M2-derived extracellular vesicles (EVs) and OC cells cocultured with M2-derived EVs. NEAT1 was found to adsorb miR-101–3p, leading to increased expression of ZEB1 and PD-L1. Both *in vitro* and *in vivo* experiments illustrated that NEAT1, delivered via EVs derived from M2 cells, stimulated the proliferation of OC cells, triggered apoptosis in CD8+ T cells, and facilitated tumor growth. Research suggests that M2-derived EVs containing NEAT1 have a tumor-promoting effect on OC through the miR-101–3p/ZEB1/PD-L1 axis (78). Yin, Wang et al. found that TAMs expressing Siglec-9 were associated with an immunosuppressive microenvironment in tumors. Blocking Siglec-9 inhibited SHP-1, an inhibitory phosphatase, leading TAMs to display an anti-cancer phenotype. Combining Siglec-9 inhibition with anti-PD-1 antibody enhanced the cytotoxicity of CD8+ T cells in tissues with abundant Siglec-9+ TAMs. These findings suggest that the presence of Siglec-9+ TAMs may independently predict poor survival outcomes and serve as a potential biomarker for PD-1/programmed death ligand-1 immunotherapy in HGSC. Further exploration of targeting Siglec-9+ TAMs for therapy is warranted (79). Brauneck et al. observed that M2 macrophages in HGSOC frequently express TIGIT, CD226, TIM-3, and LAG-3 compared to HDs. Higher TIGIT expression correlated with increased tumor grades in HGSOC, suggesting prognostic significance. Blocking TIGIT reduced the frequency of M2 macrophages, and combining TIGIT and CD47 blockade enhanced phagocytosis of (OC cells by TAMs compared to CD47 blockade alone. These findings propose a combination approach of TIGIT and CD47 blockade to enhance anti-CD47 treatment efficacy. As reported by Brauneck (80), Le et al. discovered that supernatant from M2 macrophages promoted the growth, infiltration, and motility of A2780/DDP cells. However, the TPL reversed this effect in a dose-dependent manner. Moreover, the combination treatment of TPL and DDP significantly reduced the tumor burden by inhibiting the polarization of M2 macrophages. The experiment led to a longer lifespan for mice and a decrease in CD31 and CD206 levels. Sequencing showed that DDP increased Akkermansia, whereas TPL increased Clostridium. Moreover, DDP and TPL together decreased Lactobacillus and Akkermansia. These findings suggest that M2 TAMs, invasiveness, and tolerance to DDP in epithelial EOC are significantly affected by migratory capacity, and that TPL can revert M2 macrophage polarization (73). TAMs are summarized in **Table 1**.

**Table 1:** Summary of TAMs.

Cell types	Phenotype
Inflammatory monocyte	CD14+,HLA-DR high,CD11c+,CD64+
M1 macrophage	HLA-DR+,CD68+,CD80+,CD86+
M2 macrophage	HLA-DR+,CD68+,CD163+,CD206+,CD200R
M-MDSC	CD11b+,CD33+,CD14+,HLA-DR low
G-MDSC	CD11b+,CD33+,CD15+,CD66b+,HLA-DR low

### 4.2 CAFs

In comparison to normal fibroblasts (NFs), CAFs exhibit distinctions in morphology, function, and gene expression (81). CAFs not only foster local tumor growth but also facilitate distant metastasis through diverse mechanisms. Moreover, CAFs contribute to tumor immune evasion by secreting a variety of cytokines and chemokines that impact the recruitment and function of immune cells (35, 82). Tumor cells can evade immune surveillance by establishing an immunosuppressive microenvironment. According to Wu et al., the activation of extracellular signal-regulated kinase induced by collagen type XI alpha 1 (COL11A1) prompts the translocation of p65 to the nucleus, thereby activating TGF-β3. COL11A1 overexpression in cells promotes tumorigenesis and the formation of CAFs. TGF-β3 inhibits CAF activation, whereas TGF-β3 promotes CAF activation. COL11A1 and IGFBP2 expression is upregulated in human tumors with elevated levels of TGF-β3, which correlates with reduced survival rates. These findings suggest that targeting CAFs in ovarian tumors positive for COL11A1 could be effectively achieved through an anti-TGF-β3 treatment approach. This study was conducted by Wu (2020) and Kim et al. (year not provided). This study analyzed various CAFs isolated from OC tissues and compared their gene expression profiles. The expression profile revealed that GLIS (1 Glis family zinc finger) was among the genes whose expression increased in metastatic CAFs (mCAFs).

A significant increase in both gene mRNA and protein expression was observed. Reducing GLIS1 in mCAFs significantly decreased the migratory, invasive, and wound healing abilities of OC cells. Additionally, an animal study indicated that knocking down GLIS1 in CAFs reduced peritoneal metastasis. These results imply that CAFs’ overexpression of GLIS1 enhances the migration and metastasis of OC cells, suggesting that targeting GLIS1 could be a promising therapeutic approach to inhibit OC metastasis (83). A study by Akinjiyan et al. found that DDR2 regulates the expression of POSTN in CAFs associated with OC. Furthermore, the presence of both DDR2- and POSTN-expressing CAFs resulted in a greater tumor load than the presence of CAFs lacking DDR2 and POSTN. Notably, coinjection of DDR2-deficient CAFs expressing POSTN with ovarian tumor cells led to a significant increase in tumor burden. These findings suggest that DDR2 regulates the expression of periosteal proliferative proteins through integrin B1 (integrin B1). There was a strong correlation between DDR2 expression in the tumor stroma and POSTN expression in the stromal cells of OC patients. Consequently, the regulation of OC metastasis through periosteal proliferative proteins is influenced by DDR2 expression in CAFs (84). Lin et al. reported that periostin (POSTN) enhanced integrin/ERK/NF-κB signaling through autocrine effects, resulting in polarization to M2 macrophages *in vitro*. Tumors overexpressing SKOV3 with POSTN contained more tumor-associated macrophages than did the controls. Similarly, the number of CAFs was increased in metastatic tumors derived from SKOV3 cells overexpressing POSTN. In terms of clinical relevance, POSTN expression correlated with late-stage disease and low overall patient survival. The results suggest that POSTN integrin NF κB-mediated signaling contributes to the enhancement of M2 macrophages and CAFs. This indicates that POSTN might serve as a valuable prognostic indicator and a potential target for therapeutic interventions. Akinjiyan et al. observed that the invasive capability of tumor cells decreased when exposed to CAF conditioned media (CM) lacking DDR2 or arginase-1. However, this invasive deficiency was not observed in cells consistently overexpressing arginase-1 (85). The presence of CM from DDR2-depleted CAFs with constitutive arginase-1 overexpression restored this invasion defect. Moreover, the supplementation of exogenous polyamines to CM derived from DDR2-depleted CAFs increased tumor cell invasion. DDR2-depleted CAFs exhibited reduced levels of SNAI1 protein in the arginase-1 promoter region. These findings illustrate how DDR2 regulates collagen production by modulating arginase-1 transcription, which is a crucial source of arginase activity and L-arginine metabolites in OC models (86). Studies indicate that CAFs can communicate with neighboring cells through exosomes, thereby participating in OC initiation and progression. Guo et al. treated CAFs with a microRNA-98–5p (miR-98–5p) inhibitor, isolated exosomes and cocultured them with OC cells. Finally, the impact of exosomal miR-98–5p on cisplatin resistance in OC cells was investigated. CDKC1A expression levels were greater in cisplatin-sensitive OC cell lines. CDKC1A expression was inhibited through the targeting action of miR-98–5p. Furthermore, CAF-derived exosomes containing miR-98–5p led to enhanced proliferation and cell cycle progression in OC cells. Additionally, exosome-mediated delivery of miR-98–5p promoted cisplatin resistance and downregulated CDKN1A in nude mice. These experimental findings suggest that CAF-originated exosomes with increased miR-98–5p levels promote the emergence of cisplatin resistance by suppressing CDKN1A expression in nude mice (87). Cui et al. identified that miR-630 overexpression increased FAP and α-SMA levels in NFs, inducing their transformation into CAFs. miR-630 targets KLF6, and inhibiting miR-630 or enhancing KLF6 expression mitigated EV-induced CAF activation. EVs triggered the NF-κB pathway via the miR-630/KLF6 axis. The invasion and metastasis of OVCAR8 cells were enhanced by the CM from NFs that had been pretreated with EVs. However, the promotion by NFs was partially hindered when miR-630 in EVs was downregulated. These findings suggest that miR-630 is transported into NFs through EVs, leading to the activation of CAFs and facilitating OC invasion and metastasis by inhibiting KLF6 and activating the NF-κB pathway. Our study provides insight into the mechanism underlying OC invasion and metastasis within the TME (88). Sun et al. assessed the expression of secretory leukocyte protease inhibitor (SLPI) in OC cells, tissues, CAFs, and EVs, and examined the impact of exogenous SLPI on OC cells *in vitro*. The investigation revealed a significant increase in SLPI protein expression in a subset of CAFs characterized by high FAP levels and low α-SMA expression. This upregulation correlated with higher tumor grade and decreased overall survival (OS). Notably, SLPI proteins from CAFs could be encapsulated into EVs for targeted delivery to OC cells, activating the PI3K/AKT pathway. Additionally, a strong association was observed between elevated levels of encapsulated SLPI in plasma samples from OC patients and advanced tumor stage. These findings provide evidence for the oncogenic role of EV-encapsulated SLPI secreted by CAFs in driving TACs, suggesting its potential as a prognostic biomarker for OC (89). Mo et al. identified miR-141 as an exosomal miRNA that reprograms stromal fibroblasts into pro-inflammatory CAFs, promoting metastatic colonization. Mechanistically, miR-141 targets YAP1, a key effector of the Hippo pathway, and enhances the production of GROα by stromal fibroblasts. Stroma-specific knockout (cKO) of Yap1 in a mouse model results in a microenvironment enriched for GROα and promotes tumor colonization *in vivo*, but this effect is reversed by depletion of Cxcr1/2 in OvCa cells. The results highlight the relevance of YAP1/GROα in clinical samples and propose a potential therapeutic intervention to impede the formation of pre-metastatic niches and metastatic progression in OC (90). Han et al. isolated exosomes from primary omental NFs and CAFs obtained from OC patients and assessed their impact on metastasis. Among the down-regulated miRNAs by CAF-Exo, miR-29c-3p in OC tissues was associated with patient metastasis and survival. Elevating miR-29c-3p levels significantly reduced the metastasis-promoting effect of CAF-Exo by directly targeting matrix metalloproteinase 2 (MMP2). These findings provide evidence for the significant contribution of exosomes derived from omental CAFs to peritoneal metastasis in OC, which may be partly explained by the alleviation of low levels of miR-29c-3p-mediated inhibition on MMP2 expression (91). Sun et al. identified a specific overexpression of microRNA (miR)-296–3p in EVs derived from activated CAFs. The proliferation, migration, invasion, and drug resistance of OC cells were significantly increased by the upregulation of miR-296–3p in laboratory experiments. Tumor growth was also stimulated *in vivo*. Mechanistic investigations demonstrated that miR-296–3p facilitated OC progression through direct targeting of PTEN and SOCS6 genes and activation of AKT and STAT3 signaling pathways. Elevated levels of miR-296–3p within plasma-derived EVs were strongly associated with tumorigenesis and chemotherapy resistance in OC patients. These findings offer new evidence supporting the involvement of CAF-derived EVs carrying miR-296–3p in promoting OC progression and suggest the potential of miR-296–3p encapsulated within CAF-derived EVs as a diagnostic biomarker and therapeutic target for OC treatment (92). The transfer of DNA from OC cells to CAFs was facilitated by cisplatin, as discovered by Liu and colleagues. This process induces activation of the CGAS-STING-IFNB1 pathway in CAFs, resulting in the release of IFNB1. Consequently, the resistance of cancer cells to platinum-based drugs is augmented. High levels of STING expression in the tumor stroma have been correlated with poor prognosis, while inhibition of STING expression heightens susceptibility to OC. The association between the CGAS-STING pathway and platinum drug resistance in CAFs suggests that targeting STING could represent a promising approach for combination therapy in OC, offering potential opportunities for enhancing treatment outcomes (93). Jiang et al. obtained CAFs and NFs from ovarian tumors and healthy ovaries, respectively. Overexpression of miR-1290 in CAFs significantly increased viability, DNA synthesis, and cell invasion in OC cells, and altered the expression of epithelial-mesenchymal transition (EMT) markers in OC cells. Finally, overexpression of miR-1290 in CAFs increased tumor growth in a nude mouse xenograft tumor model. These findings suggest that the miRNA/mRNA axis in OC CAFs may regulate the proliferation and invasion of OC cells through the Akt/mTOR pathway (94).

Moreover, cytokines derived from CAFs also exert significant biological effects. Thongchot et al. assessed cancer cell migration using the Transwell migration assay and investigated the role of interleukin-8 (IL-8) in OC cell migration, along with its mechanistic connection to autophagy. Additionally, the pro-migratory impact of IL-8 was mitigated by pharmacologically inducing autophagy with rapamycin or metformin. Neutralizing anti-IL-8 antibodies counteracted the inhibitory effect of OVCAFs-CMMPs. The experimental results argue for the involvement of IL-8 released by CAFs in ovarian tumors. Autophagy in the ovarian TME is inhibited to promote cancer cell migration (95). Ji et al. uncovered that IL-8 secretion by CAFs can trigger the activation of normal ovarian fibroblasts (NFs) through diverse signaling pathways. Moreover, IL-8 was found to enhance the malignant growth of OC cells in animal models and increase their resistance to cisplatin (CDDP), evidenced by elevated IC50 values for OC cells. Further investigations revealed that IL-8 promotes cancer cell stemness induction via Notch3, with a positive correlation observed between elevated IL-8 levels in ascites and Notch3 expression in OC tissues. In essence, the activation of Notch3-mediated signal transduction through IL-8 secretion from CAFs and cancer cells significantly contributes to promoting stemness in human OC. These findings may offer a novel approach for treating OC (96). Jin et al. reported that collapsin response mediator protein-2 (CRMP2) from CAFs is a key regulator mediating these cellular events in ovarian cancer (OvCA). *In vitro* investigations utilizing recombinant CRMP2 (r-CRMP2) demonstrated that this protein stimulates OvCA cell proliferation by activating the hypoxia-inducible factor (HIF)-1 alpha-glycolytic signaling pathway, invasion, and migration. Analysis of patient samples revealed abundant expression of CRMP2 in OvCA, strongly correlated with cancer metastasis and an unfavorable prognosis. Inhibition of CRMP2 in CAFs by neutralizing antibodies significantly ameliorated tumors in mice *in vivo*. Our findings provide new insight into TME-based OC treatment (97). Dai et al. reported that the migration of OC cells cocultured with CAFs was significantly enhanced. In addition, the density of CAFs in metastatic sections was greater than that in primary OC primary tumor sites. We found that co-culture of SKOV3 with recombinant human stromal-derived factor-1α (SDF-1α) significantly inhibits cisplatin-induced cytotoxicity and apoptosis in a dose- and time-dependent manner, with the CXCR4 antagonist AMD3100 blocking this effect. These results suggest CAFs may contribute to malignant OC metastasis by promoting tumor cell migration. In OC, CAFs’ resistance to cytotoxic drugs may be mediated through SDF-1α/CXCR4 signaling (98). Hu et al. identified a subset of CAFs expressing INHBA as significant promoters of tumor growth and immune suppression in OC. Reanalyzing patient samples revealed metastatic tumors with high INHBA(+) CAF levels also had increased regulatory Tregs. Co-culturing human ovarian CAFs with T cells showed direct contact between INHBA(+) CAFs and T cells is crucial for promoting Treg differentiation. This involves activating autocrine PD-L1 expression in CAFs through SMAD2-dependent signaling triggered by INHBA/recombinant activin A, ultimately facilitating Treg differentiation. These findings highlight the therapeutic potential within the INHBA(+) subset for advanced OC treatment, especially considering its typically poor response to immunotherapy.

In summary, the above studies illustrate how CAFs contribute to cancer advancement through the secretion of growth factors, cytokines, and chemokines, as well as ECM degradation. Additionally, CAFs can release taste-promoting cytokines locally; thus, aiding in the spread of OC cells. Moreover, CAFs promote immune evasion by upregulating immune checkpoint ligands and immunosuppressive cytokines, hindering the infiltration of antitumor CD8+ T lymphocytes, and triggering antitumor responses by interacting with other immune cells. Increasing evidence suggests that CAFs mediate chemotherapy resistance in OC, which supports the role of CAFs as promising therapeutic targets for treating OC.

### 4.3 MDSCs

MDSCs constitute a highly diverse array of immune cells emerging in various physiological and pathological contexts, notably in inflammatory settings like cancer, infection, trauma, and autoimmune diseases (99). Primarily originating from the bone marrow, MDSCs include immature myeloid cells, including early granulocytes, monocytes, and other myeloid precursors, which typically mature into immune cells such as neutrophils, monocytes, and macrophages under normal conditions (100). However, in inflammation or the TME, the differentiation of MDSCs is disrupted by signaling molecules such as cytokines and growth factors, resulting in their accumulation. MDSCs suppress immune responses by inhibiting T-cell activation and proliferation, promoting regulatory Treg development and function, and modulating other immune cell activities (101). Their accumulation is a key mechanism enabling tumors to evade immune surveillance and fuel tumor growth. MDSCs directly support tumor cell survival and dissemination through immunosuppression and also contribute to angiogenesis and TME alterations (102). Furthermore, MDSCs indirectly facilitate tumor development by promoting tumor cell invasiveness and metastasis (103).

MDSCs consist of two main groups of cells, mononuclear MDSC (M-MDSC) and polymorphonuclear MDSC defined as CD11b+Ly6ChighLy6G- cells and CD11b+Ry6ClowLy6G- cells, respectively (104); therefore, individuals equivalent to M-MDSCs are defined as CD33+CD14+HLA-DR -/low CD15 - cells, while PMN-MDSCs are defined as CD13+CD14-CD15+ or CD33+CD14-CD66b+ cells. Abundant M-MDSCs have been observed in the peripheral blood and ascites of OC patients, with their accumulation and inhibitory activity primarily attributed to ascites-derived IL-6 and IL-10, along with downstream STAT3 signals (105). Okta et al. found a correlation between elevated M-MDSC quantities in tumors and the progression to advanced stages and higher grades of EOC. They also noted differences in immunosuppressive patterns between EOC patients and healthy donors, with a significant increase in ARG/IDO/IL-10-expressing M-type and PMN-MDSCs in the blood of EOC patients. The accumulation of these subpopulations positively correlated with TGF-β and ARG1 levels in plasma and peritoneal fluid (PF). In OC patients, prolonged survival was significantly associated with reduced levels of circulating and intratumoral M-MDSCs, indicating their potential clinical significance (106). Horikawa et al. observed that treatment with anti-VEGF led to the up-regulation of granulocyte-monocyte colony-stimulating factor (GM-CSF), promoting the migration and differentiation of MDSCs while inhibiting the proliferation of CD8+ lymphocytes. Targeting GM-CSF improved therapy efficacy by reducing MDSC infiltration and increasing CD8+ lymphocytes. Additionally, enhanced expression of GM-CSF was found in bevacizumab-resistant patients, suggesting that GM-CSF plays a role in recruiting MDSCs to suppress tumor immunity induced by hypoxia caused by anti-VEGF therapy. Targeting GM-CSF could overcome resistance to this therapy for OC (107). In their study, Li et al. identified various genes that were differentially expressed in EOC cells. Notably, when EOC cells were cocultured with MDSCs, a significant increase in the expression level of colony-stimulating factor 2 (CSF2) was observed. Furthermore, successful depletion of CSF2 has been accomplished in these cells. Interestingly, the downregulation of CSF2 expression effectively counteracted the increase in EOC cell stemness induced by MDSCs. Additionally, inhibition of p-STAT3 also led to a significant reversal in the promotion of EOC cell stemness caused by MDSCs. In addition, CSF2 expression levels correlated with EOC clinical stage. These findings suggest that MDSCs enhance EOC cell stemness by inducing the CSF2/p-STAT3 signaling pathway. Enhancing the effectiveness of conventional treatments could be achieved by focusing on MDSCs or CSF2 as viable targets (108). Okla et al. discovered that M-MDSCs exhibited greater PD-L1 expression than MO/MA in both blood and ascites samples. The expression of PD-L1 was notably elevated in ICs compared with that in TCs, although PD-L1+ TC levels were more prominent in endometrioid and mucinous tumors. Furthermore, there was a direct association between the levels of circulating sPD-L1 and the numbers of PD-L1+ M-MDSCs and PD-L1+MO/MA in the bloodstream. Neither PD-L1 nor sPD-L1 served as prognostic indicators for overall survival (OS). The experimental findings suggested that while PD-L1 may not predict OC outcomes, its upregulation indicated immune impairment without prognostic implications. Moreover, PD-L1+ myeloid cells in blood correlated positively with sPD-L1, suggesting sPD-L1 might serve as a non-invasive surrogate marker for immune surveillance of PD-L1+ myeloid cells in OC (109). McGray et al. proposed exploring other therapeutic combinations to enhance CD8+ T cell function using the primary/booster vaccine platform. They observed moderate tumor control enhancement with CD27 agonists or antibody-mediated granulocyte depletion post-vaccination, while adding anti-PD-1 therapies further improved treatment outcomes. These findings underscore the potential of immunotherapies with well-defined mechanisms of action as a basis for identifying combination approaches for treating OC (110). pi et al. reported that high expression levels of mTORC2 were associated with shorter survival in EOC patients, whereas mTORC1 was unrelated to patient prognosis. Azd2014 inhibits the mTOR signaling pathway in OC cells and suppresses cell proliferation. Azd2014 specifically reduces the migration and aggregation of MDSCs in the peritoneal tissue of EOC patients and the aggregation of MDSCs in EOC peritoneal tissue but not in the spleen. In addition, AZD2014 treatment after cisplatin chemotherapy delayed the recurrence of EOC. Our findings suggest that high mTORC2 expression in EOC portends a poor prognosis. Notably, in tumor-bearing mice, AZD2014 reduced MDSC accumulation and delayed tumor growth and recurrence. (Pi 34560229). Yang et al. reported that there was an increase in the proportion of MDSCs in the peripheral blood of obese mice. Additionally, IL-6 significantly enhanced the expression levels of S100A8 and S100A9 in MDSCs. Furthermore, the infiltration of MDSCs into OCs was directly related to the level of IL-6 expression. The levels of IL-6 observed in OC tissues were positively associated with the expression levels of S100A8 and S100A9. Finally, LMT28 can inhibit tumor growth by suppressing IL-6. Obesity promotes immune evasion and metastasis in OC by upregulating IL-6 and promoting the expression of the MDSC-associated immunosuppressive genes S100A8 and S100A9 (111). Chen et al. reported that Ankrd22 knockdown increased CCR2 expression in CD11b+ Ly6G+ Ly6Clow cells and the immunosuppressive activity of PMN- MDSCs. In a mouse model of tumor xenografts, CD11b+ Ly6G+ Ly6Clow cells organized biochemically from Ankrd22-/- mice significantly enhanced the proliferation of OC cells. RNA sequencing revealed a significant increase in the expression of Wdfy1 in these cells. Furthermore, a potential small molecule compound activating ANKRD22 was found to weaken the immunosuppressive activity of Ankrd22+/+ PMN-M MDSCs. These findings suggest ANKRD22 as a promising target for reversing the immunosuppressive effects of PMN-M DSCs (112). Wang et al. explored the influence of METTL3 on IL-1β secretion and inflammasome activation in the context of OC. The study observed increased OC cell growth in Mettl3-cKO mice, along with a transition in macrophage polarization from reduced M1 to increased M2 during OC progression. Additionally, Mettl3 deficiency in myeloid cells resulted in increased secretion of CCL2 and CXCL2 in peritoneal lavage fluid. Importantly, the deletion of Mettl3 amplified IL-1β secretion induced by viable ID8 cells. These insights shed light on how METTL3-mediated m6A methylation affects the immune response against OC (100).

### 4.4 DCs

DCs are vital to the human immune system, serving as essential mediators in capturing, processing, and presenting antigens, and acting as a crucial bridge between innate and adaptive immunity (113). These cells are distributed throughout multiple tissues, organs, the blood, and lymphatic systems. The primary function of DCs is to recognize and capture antigens, presenting them to T cells to initiate a T-cell-mediated immune response (114, 115). After capturing antigens, DCs migrate from peripheral tissues to lymph nodes, undergoing maturation and exhibiting elevated levels of major histocompatibility complex (MHC) molecules and costimulatory molecules. The critical role of DCs in initiating and regulating the immune response highlights their importance as a research target in immunotherapy. Modifying the activity and function of DCs may lead to new vaccine developments and therapeutic approaches to effectively combat cancer and other immune-related diseases (116). Additionally, DCs play a significant role in studying autoimmune diseases, infectious diseases, and transplant rejection. Gao et al. found that high expression of Growth differentiation factor-15 (GDF-15) was linked to the infiltration of immune DCs in immunoreactive high-grade plasmacytoid carcinoma. Moreover, GDF-15 inhibited the maturation of DCs. Overexpression of CD44 in DCs inhibited GDF-15 effects on DC synapse length and number. The inhibitory effect of GDF-15 on CD11c, CD83, and CD86 expression was attenuated in DCs overexpressing CD44, and this inhibitory effect was further enhanced in DCs knocked down for CD44, while CD44 overexpression suppressed the inhibitory effect of GDF-15 on DC migration. These findings suggest that GDF-15 may inhibit the function of CD44 in DCs by interacting with it, thereby promoting immune escape from OC (117). According to a study by Luo et al., it was discovered that the Th17-DC vaccine positively impacted the TME by increasing the presence of Th17 T cells and remodeling the bone marrow microenvironment. This resulted in an improved survival rate for mice compared to those treated with a cDC vaccine. While immune checkpoint blockade (ICB) showed limited effectiveness against OC, the administration of a Th17-inducing dendritic cell (DC) vaccination sensitized OC cells to PD-1 ICB, effectively overcoming IL-10-induced resistance. The efficacy of the Th17-DC vaccine, either alone or in combination with ICB, was found to be mediated by CD4 T cells rather than CD8 T cells, highlighting the potential benefits of utilizing biologically relevant immunomodulators like the Th17-DC vaccine in OC therapy as a means to remodel the TME and enhance clinical response to ICB therapy (118).

### 4.5 NK cells

NK cells are important immune cells that mediate tumor immunosurveillance (119). The recognition and elimination of target cells by NK cells do not require prior exposure to pathogens, especially those infected with viruses and mutated tumor cells (120). Immunosurveillance relies heavily on the pivotal contribution of NK cells, responding rapidly and directly killing cells that do not express sufficient amounts of MHC I molecules (121, 122). This recognition mechanism allows NK cells to bypass the immune evasion strategies of certain pathogens and tumor cells. In addition to their direct killing function, NK cells can influence and regulate other immune cells by secreting cytokines to promote an immune response (119, 123). Meer et al. found that N-803 also enhances HPC-NK cell-mediated leukemia killing. Treatment of OC spheroids with HPC-NK cells and N-803 increased target killing. In immunodeficient mice harboring human OCs, the binding of N-803 to whole human immunoglobulin supported the persistence of HPC-NK cell N-803 binding to whole human immunoglobulin, preventing Fc-mediated HPC-NK cell depletion. In addition, the combination therapy reduced tumor growth. These results suggest that N-803 is a promising agent for enhancing the proliferation and function of HPC-NK cells both in laboratory settings and in animal models. Integrating N-803 into HPC-NK cell therapies could potentially improve the efficacy of cancer immunotherapy (124). Meer et al. noted that gemcitabine did not affect the characteristics or function of HPC-NK cells, though OC cells showed increased expression of NK cell activating ligands and death receptors. While pretreatment of OC cells with gemcitabine did not enhance HPC-NK cell function, the combination of HPC-NK cells and gemcitabine was effective in killing OC cells *in vitro*. Additionally, this combination therapy decreased tumor growth in OC mouse models. These findings support that the joint application of HPC-NK cells and gemcitabine enhances the destruction of OC cells both in the laboratory and *in vivo* environments. This supports further exploration of this treatment strategy in patients with recurrent OC (125). Using a murine experimental model of advanced EOC, Vloten et al. reported that Orf virus (OrfV) is a therapeutic agent. It was demonstrated in experiments with knockout mice that OrfV therapy requires classical type 1 dendritic cells (cDC1s). In addition, cDC1s control antitumor NK and T-cell responses, thereby mediating the antitumor efficacy of OrfV. Primary tumor resection is a commonly used treatment for human patients and is effectively combined with OrfV to achieve optimal therapeutic outcomes. achieves optimal therapeutic effects. Furthermore, cDC1s were associated with NK cells in human OC, and intratumoral NK cells were positively correlated with survival. These findings demonstrate the potential of OrfV as an NK-stimulating immunotherapy for the treatment of advanced OC (126). Fraser et al. discovered that the expression of genes related to cytotoxicity and signaling pathways decreased in NK cells isolated from the ascites of OC patients. Similarly, NK cells obtained from treated healthy donors also displayed downregulation of genes involved in cytotoxic pathways. These findings indicate that both ascites and CA125 impede the anti-tumor activity of NK cells by suppressing gene expression responsible for their activation and ability to kill cancerous cells at the transcriptional level. This study provides a deeper insight into how ascites inhibits NK cell function and suggests potential strategies for reactivating these immune cells as part of OC immunotherapy (127). Raja et al. discovered that Protein phosphatase 4 (PP4) inhibitors or agents that knock down PPP4C in combination with carboplatin triggered inflammatory signaling. Inhibiting PP4 results in reduced CD8 T-cell migration. Co-culturing NK-92 cells and OC cells via PPP4C or PPP4R3B suppression enhances NK cells’ ability to eliminate OC cells. In an immunocompetent mouse model, stable knockdown of PP4C significantly restrains tumor growth. These findings propose that PP4 inhibitors can stimulate inflammatory signaling and enhance immune cell response efficacy. Hence, further investigation into PP4 inhibitors’ use in combination with chemoimmunotherapy for treating OC is justified (128). Luo et al.’s study illustrated that expanded natural killer cells (eNK-EXO), loaded with cisplatin, sensitize drug-resistant OC cells to cisplatin’s antiproliferative effects. Additionally, eNK-EXO can activate NK cells within the immunosuppressive TME, and researchers have explored the underlying mechanism involved. In conclusion, the inherent antitumor activity of eNK-EXO suggests their potential as therapeutic agents for OC. Furthermore, utilizing eNK-EXO as carriers for cisplatin can enhance the effectiveness of drugs against drug-resistant OC cells. Additionally, eNK-EXO has shown promise in reversing the immunosuppressive effects of NK cells. These findings present significant prospects for the clinical application of eNK-EXO in treating OC and pave the way for further investigations into its efficacy in other solid tumor treatments (129). Steitz et al. discovered that tumor-associated NK cells induced TRAIL-dependent apoptosis in mesothelial cells upon encountering activated T cells. Moreover, the upregulation of TRAIL expression in NK cells and the enhanced cytotoxicity to mesothelial cells were primarily driven by T cell-derived TNFa. Importantly, apoptotic mesothelial cells were observed in the peritoneal fluid of HGSC patients. Conversely, HGSC cells exhibited resistance to TRAIL, indicating a cell type-selective killing effect of NK cells. The findings support a synergistic role of T cells and NK cells in breaching the mesothelial cell barrier in HGSC patients (130).

### 4.6 T cells

T lymphocytes play a pivotal role in the body’s immune response against tumors, capable of combatting both pathogens and tumor cells (131) and identifying processed antigenic fragments through the T-cell receptor (TCR) (132). The main subsets of T cells include CD4+ T cells and CD8+ T cells. CD4+ T cells primarily regulate the immune response by releasing various cytokines to activate or suppress other immune cells. CD8+ T cells, also referred to as CTLs, possess the ability to directly eliminate virus-infected cells or tumor cells (133).

Chen et al. observed a notable rise in TIGIT expression among CD4+ Tregs. Furthermore, the application of anti-TIGIT therapy led to a decline in the CD4+ Tregs proportion, with no discernible effect on CD4+ and CD8+ T cells or NK cells. Additionally, TIGIT inhibition resulted in a decrease in the level of immunosuppression induced by CD4+ Tregs. Survival analysis indicated that anti-TIGIT treatment notably enhanced the survival rate of mice with OC. These findings suggest that TIGIT contributes to enhancing the response to CD4+ Tregs and mediating immunosuppression within the OC model. Consequently, inhibiting TIGIT could be a viable therapeutic approach for patients with OC (134). Silveira et al. discovered that P-MAPA treatment elevated the levels of TLR2 and TLR4 in OC while decreasing the count of regulatory Tregs. Furthermore, the interaction of P-MAPA with IL-12 notably augmented the population of CD4+ and CD8+ effector cells in draining lymph nodes. Concerning inflammatory mediators, P-MAPA raised the levels of the proinflammatory cytokine IL-17, whereas P-MAPA + IL-12 increased the levels of IL-1β. These findings suggest that P-MAPA upregulates TLR2 and TLR4 signaling while attenuating tumor immunosuppression. Furthermore, P-MAPA, in combination with IL-12, enhances antitumor immune responses, opening a new therapeutic avenue in the fight against OC (135). Werehene et al. observed that under chemosensitive conditions, the secretion of sEV-pGSN decreased. This led to an increase in IFNg release by T cells, resulting in decreased intracellular glutathione (GSH) production and increased susceptibility of chemotherapy-sensitive cells to cis-dichloroplatinum (CDDP)-induced programmed cell death. In cases of chemotherapy resistance, OC cells showed increased secretion of sEV-pGSN, inducing apoptosis in CD8þ T cells. Consequently, IFNg secretion decreased, leading to elevated GSH production. These findings suggest that sEV-pGSN plays a role in suppressing immune surveillance and regulating GSH production, contributing to the development of chemoresistance in OC (136). Desbois et al.’s research identified two distinct features of T-cell exclusion from tumors: 1) loss of antigen presentation by tumor cells; and 2) upregulation of TGFβ and stromal activation. Moreover, TGFβ significantly inhibits T-cell infiltration. *In vitro* experiments demonstrated that TGFβ reduces MHC-I expression in OC cells. Additionally, TGFβ stimulates fibroblasts and enhances ECM production, potentially forming a physical barrier to impede T cell entry. These findings propose that targeting TGFβ could be a promising strategy to overcome immune rejection by T cells and enhance the clinical efficacy of cancer immunotherapy (137). McCaw et al. reported that the class I HDAC inhibitor entinostat upregulated pathways and genes associated with the cytotoxic function of CD8+ T cells while downregulating the expression of myeloid-derived suppressor cell chemoattractant factors. The inhibitory potential of regulatory T cells in tumors and associated ascites was significantly diminished, leading to a reversal in the CD8-Treg ratio. These findings illustrate that class I HDAC inhibition fosters intratumoral CD8 T cell activation by disrupting the suppressive network in the EOC TME. Consequently, class I HDAC inhibition may render advanced EOC susceptible to immunotherapeutic modalities (138). Sima et al. uncovered that the absence of TG2 in mice resulted in an augmentation of cytotoxic responses of CD8+ T cells specific to tumor antigens present in ascites. Additionally, the depletion of CD8+ T cells hastened the accumulation of ascites in TG2-/- mice. CD8+ T cells obtained from tumor-bearing TG2-/- mice exhibited characteristics associated with effector T cells. Mechanistically, the absence of TG2 amplifies signals that facilitate the activation of T cells. Intraperitoneally growing cancer cells produced a stronger immune response to TG2 deletion. Furthermore, TG2 expression in the stroma but not in the tumor was indirectly correlated with the number of tumor-infiltrating lymphocytes. The results demonstrated that the TME in TG2-/- mice has a reduced tumor load, enhanced T-cell activation and effector function, and loss of immunosuppressive signals. This leads to the hypothesis that TG2 is an attenuator of antitumor T-cell immunity and a novel immunomodulatory target (139). Muthuswamy et al. reported high expression of CXCR6 on chemokine receptors in OC patients. The analysis revealed a connection between CXCR6 and increased CD103 levels, correlating with enhanced patient survival. Furthermore, CXCR6 acts as an exclusive marker for tumor-specific memory CD8+ T cells residing within the tumor rather than circulating in the bloodstream. Elimination of CXCR6 in these specific CD8+ T cells leads to reduced retention within tumor tissues, resulting in a weakened resident memory response and compromised control over OC. These results emphasize the vital role of CXCR6 in immune surveillance and OC management by promoting the retention of resident memory T cells within tumor tissue. Future studies should explore utilizing CXCR6 to improve resident memory responses against cancer (140). Tsuji et al. conducted a thorough analysis at the single-cell level and proposed a model in which CD103TCF1+ recirculating T cell precursors differentiate in response to tumor antigen recognition, underscoring the significant anti-tumor function of CD103+ TRM cells in OC (141). Kamat et al. identified elevated levels of CCL23 in both ascites and plasma samples from patients diagnosed with high-grade plasmacytoid OC (HGSC). These increased levels were associated with increased expression of exhaustion markers CTLA-4 and PD-1 on CD8+ T cells in tissues exhibiting higher CCL23 levels and macrophages. Through *in vitro* experiments, it was demonstrated that CCL23 prompts the upregulation of immune checkpoint proteins on CD8+ T cells by phosphorylating GSK3β. These results underscore the role of macrophage-derived CCL23 in shaping the immunosuppressive TME in OC, promoting a depleted T cell phenotype (142). Zhu et al. reported that OC tissues had greater expression of Rab8a, Hsp90a, and Il6 than neighboring normal tissues. IL-6 levels were correlated with the number of LC3+ EVs in ascites, and the percentage of HSP90α+ LC3+ EVs and the ROMA index of patients were positively correlated. In addition, LC3+ EVs induced elevated IL-6 production by CD4+ T cells, which was inhibited by anti-HSP90α or anti-TLR2. These findings demonstrate the associations of LC3+ EV levels and the percentage of HSP90α+ LC3+ EVs with elevated IL-6 in the ascites of EOC patients. HSP90α on human EOC LC3+ EVs stimulates IL-6 production by CD4+ T cells via TLR2 (143). In their study, Zhang et al. detected a specific group of tissue-resident memory T cells (Trm) characterized by the presence of both TIM-3 and CXCL13 markers within EOC samples. Notably, compared with other patients, high-grade plasmacytoid EOC patients with TIM-3-positive Trm cells experienced significant improvements in OS. Moreover, the presence of CXCL13-positive CD8-positive T cells demonstrated a strong association with positive responses to anti-PD1 ICIs among patients, suggesting that combining PD-1 blockers with agents targeting TIM-3 could reactivate anticancer immunity against EOC (144). Another study conducted by Vlaming et al. revealed an interesting subset of exhausted CD8+ TNFRSF1B+ T cells linked to disease progression in OC patients. Their findings from both laboratory experiments and analyses of OC patients consistently supported the notion that increased expression levels of TNFRSF1B on activated CD8+ T cells corresponded to increased clinical malignancy levels and poorer prognoses. Furthermore, the inhibition of TNFRSF1B led to a notable modification of the immune microenvironment in an OC mouse model, resulting in suppressed tumor growth. These findings highlight the potential clinical significance of targeting TNFRSF1B for immunotherapy and enhance our understanding of the factors contributing to the limited success observed in OC immunotherapeutic approaches (145). Yakubovich et al. reported that cancer cells that migrated into/out of tumors possessed more mesenchymal stromal cells than those that exited and deserted tumors. Furthermore, high LGALS3 expression was associated with EMT *in vivo*. Significantly, CD8+ T cells displayed increased expression of LAG3, a marker of T cell exhaustion. These findings suggest that the EMT process in OC cells facilitates interaction between cancer cells and T cells through the LGALS3 - LAG3 pathway, potentially leading to a decrease in T cell presence within tumor infiltrates and thereby suppressing the immune response against tumors (146).

### 4.7 Tregs

Tregs, a subset of immune-suppressing cells, play a pivotal role in maintaining immune system equilibrium and preventing autoimmune diseases. They regulate the activity of other immune cells through both contact-dependent and cytokine-mediated mechanisms (147). Dysregulation of Tregs is associated with the onset of various conditions, including autoimmune diseases, allergic reactions, and immune evasion in tumors (148). When activated by their environment, Tregs can dampen the anti-tumor immune response by releasing inhibitory cytokines like IL-10, IL-35, and TGF-β; thus, fostering tumorigenesis (149, 150). Tregs are distinguished by the expression of CD25 and Foxp3, a key marker crucial for their suppressive function. Xu et al. reported that after activation of TLR8 signaling in CD4+ Tregs, the proliferation of naive CD4+ T cells was greater than that in controls. Moreover, glucose uptake and glycolysis in TLR8-activated CD4+ Treg cells were decreased. In addition, TLR8 signaling downregulates the mTOR pathway in CD4+ Tregs. Pretreatment of CD4+ Tregs with 2-deoxy-d-glucose (2-DG) and Schisandra chinensis also reduced the inhibition of Teff proliferation. There were no significant differences between CD4+ Tregs pretreated with 2-DG and those pretreated with pentaphosphatoflavone prior to TLR8 signaling activation and those treated with inhibitors alone, demonstrating that TLR8-mediated reversal of the inhibitory effect of CD4+ Tregs on the microenvironment of cocultured OC cells is causally related to glucose metabolism (147). Shan et al. explored the presence of HVEM in the peripheral blood of OC patients and analyzed the proportion of CD4+CD25+Foxp3 positive Tregs cells using flow cytometry. They also established OC cell lines with varying levels of HVEM expression. Moreover, it was discovered that overexpressing HVEM enhanced the production of IL-2 and TGF-β1 cytokines, activated STAT5, and increased Foxp3 expression, ultimately resulting in an increase in Treg positivity rate. These findings provide experimental evidence elucidating how HVEM expression in OC cells can upregulate Tregs through the STAT5/Foxp3 signaling pathway, offering insights into potential clinical strategies for treating OC (151).

## 5 The potential of antitumor therapy on the immune microenvironment of OV

### 5.1 Chemotherapy

Chemotherapy is a widely employed medical treatment for cancer, involving the use of one or more chemical agents to eradicate or curb the proliferation and division of cancer cells. These chemotherapeutic agents function by damaging the DNA of cancer cells, obstructing crucial phases of cell division, suppressing necessary hormones or signals for cell growth, and facilitating the programmed death of the cancer cells themselves. Given that cancer cells often divide more rapidly than normal cells, chemotherapeutic agents are particularly effective at targeting these swiftly multiplying cells, although they may also impact other rapidly dividing healthy cells in the body, such as those in hair follicles, blood, and the digestive tract, leading to various side effects (152). Chemotherapy is critical in treating OC, with systemic administration being typical, allowing the drugs to circulate through the bloodstream and target both the primary tumor and any microscopic metastatic foci elsewhere in the body (153). Treatment usually involves a combination of drugs, predominantly those containing platinum and paclitaxel, which impede tumor growth by various mechanisms that damage the DNA of cancer cells; thus, preventing their replication and division. Chemotherapy may serve as a primary treatment (neoadjuvant or postoperative adjuvant chemotherapy) to lessen tumor size and reduce recurrence risk, or as palliative care for advanced or recurrent OC to extend survival and enhance the quality of life. Despite chemotherapy’s role in treating OC, its side effects and the impact on patient quality of life must be considered. Common side effects include nausea, vomiting, hair loss, fatigue, low white blood cell count, and anemia. Thus, the selection and management of chemotherapy regimens should consider the tumor characteristics, overall patient condition, and patient preferences. The influence of TME on the therapeutic outcomes of chemotherapy has been thoroughly investigated. Immune cells within tumors can suppress growth by disrupting immune regulatory tumor cells, yet they may also foster tumor resistance to treatment by affecting tumor immunogenicity and selecting tumor clones that contribute to immune evasion (154). Furthermore, immune cells in the TME play a dual role in cancer development and metastasis. Cells such as Type 1 helper T cells (Th1), cytotoxic T lymphocytes (CTL), and natural killer cells (NK cells) contribute to an immune-stimulating environment. Conversely, the regulatory cells of the TME, including Type 2 helper T cells (Th2), TAMs, regulatory T cells (Tregs), and myeloid-derived suppressor cells (MDSCs), create an immunosuppressive environment and are linked with adverse outcomes (155). These cells either support tumor eradication or promote tumor escape by removing immunogenic tumor cells or modifying tumor immunogenicity (156). Additionally, chemokines and cytokines are significant components of the tumor immune microenvironment (TIME), playing a pivotal role in balancing oncogenic and anti-tumor immune responses. The intricate interactions between cancer cells and immune niches influence immunotherapy and various other anti-cancer treatments. With advancements in personalized medicine and targeted therapeutic approaches, integrating chemotherapy with other treatments like targeted therapy and immunotherapy offers more options and hope for OC patients. It also highlights that combining agents targeting TME and chemotherapeutics may overcome drug resistance and yield synergistic effects.

### 5.2 Radiotherapy

Radiotherapy is a medical procedure that employs high-energy radiation, such as X-rays, gamma rays, or proton beams, to address cancer and certain non-cancerous conditions. This method functions by damaging the DNA of cancer cells, inhibiting their ability to divide and multiply, and ultimately resulting in their death. Radiotherapy is typically localized to the tumor area, which minimizes harm to the adjacent healthy tissue. It can be applied as an independent treatment or combined with other therapeutic strategies, such as surgery, chemotherapy, or immunotherapy, to develop a comprehensive cancer treatment plan tailored to the specific objectives and modalities of treatment. The primary limitation of radiotherapy in treating OC lies in its general indication for localized conditions, whereas OC frequently affects multiple locations on the peritoneal surface and within the abdominal cavity. Furthermore, the tissues within the abdominal cavity are particularly prone to radiation-induced damage, which restricts the safe dosage of radiation that can be administered (157). Therefore, radiotherapy, such as palliative treatment against a single residual tumor or treatment for the recurrence of OC, is mainly used in specific cases of OC, especially if multiple lines of chemotherapy are used. Nevertheless, radiotherapy continues to be an option for selected OC patients. In advanced stages of OC, radiotherapy can alleviate pain, control bleeding, or manage other symptoms; thus, enhancing the quality of life for patients. Recent advancements in radiotherapy techniques, such as intensity-modulated radiotherapy (IMRT), stereotactic radiotherapy (SBRT), and proton therapy, have significantly enhanced the precision and safety of treatments. These improvements may broaden the application of radiotherapy in OC treatment in the future.

### 5.3 Immunotherapy

Immunotherapy has emerged as a significant treatment modality for tumors in recent years (158). Rather than directly targeting tumor cells, immunotherapy engages the body’s immune system to recognize and eliminate tumors. This category includes ICIs, immunomodulators, cancer vaccines, and cellular therapies like CAR-T therapy. ICIs are particularly crucial as they enhance the immune response by blocking checkpoint proteins within the immune system, such as PD-1, PD-L1, and CTLA-4. These proteins typically maintain immune balance and prevent the immune system from attacking normal cells, but many cancer cells exploit these mechanisms to evade immune detection and destruction. By inhibiting these checkpoints, the suppression is removed, allowing the immune system to target cancer cells effectively.

The use of immunotherapy in managing OC is increasing, although its role is less defined compared to other cancers due to the immunosuppressive microenvironment of OC, which hampers the ability of immune cells to infiltrate and eliminate cancer cells. However, initial clinical trials and studies have indicated benefits for certain OC patients, particularly those who express PD-L1 or have a high mutational burden. Research is also ongoing in utilizing cancer vaccines and cellular therapies in OC treatment. These vaccines aim to prime the immune system to recognize and attack cancer cells bearing specific antigens. While still in the early stages for OC, immunotherapy has shown promise and offers an alternative for patients who respond poorly to conventional treatments like surgery and chemotherapy. As understanding of the immune microenvironment of OC deepens and immunotherapy strategies are optimized, it is anticipated that more effective treatment options will be provided and outcomes for OC patients will improve.

#### 5.3.1 PD-1 and PD-L1 inhibitors

**Figure 2:**
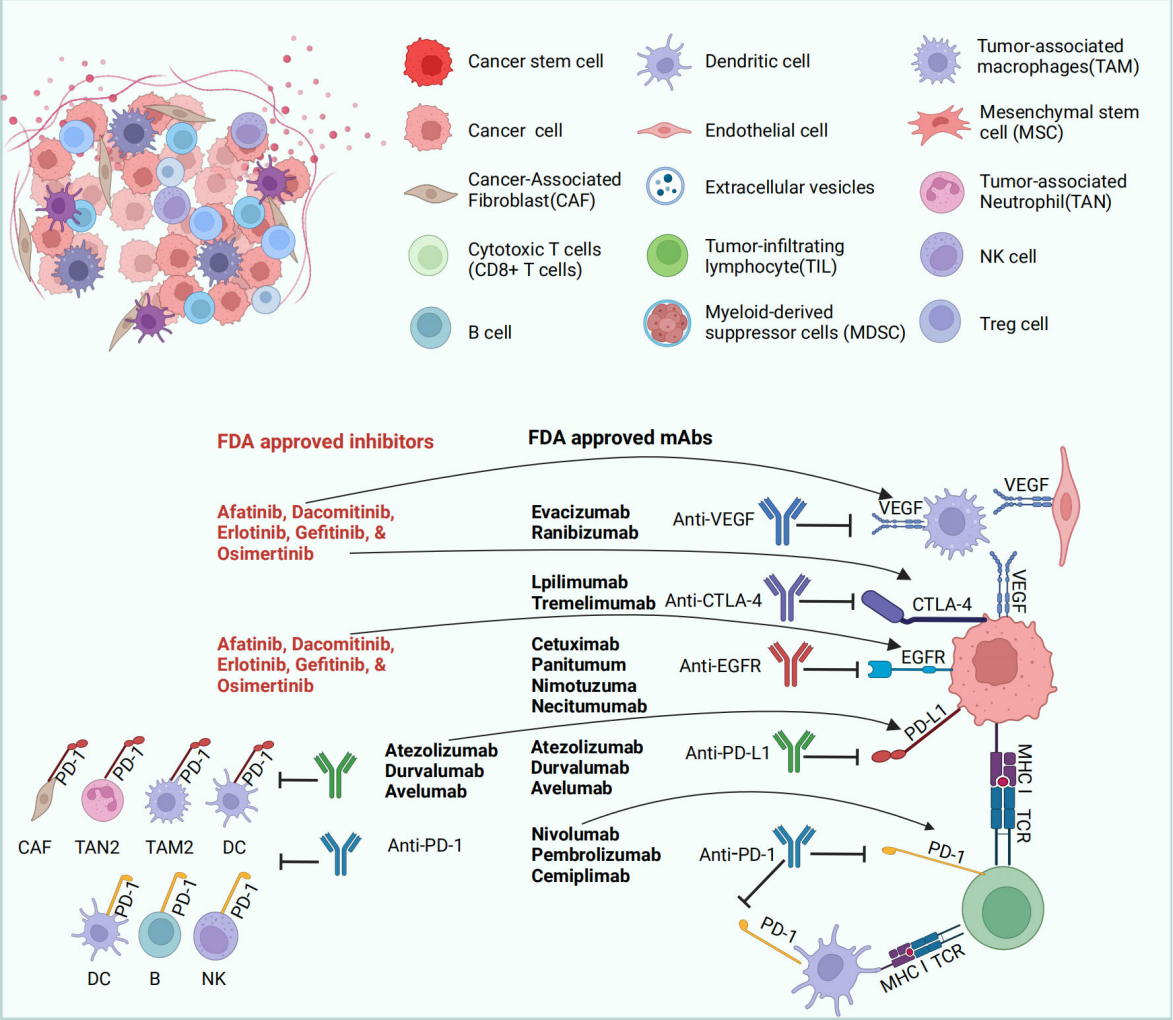
PD-1/PD-L1 interaction-mediated T-cell inhibition. Many mechanisms, such as genomic aberrations, oncogenic transcription factors and pathways, and post-translational regulation and transport, are involved in the regulation of PD-L1 expression. In addition, anti-PD-1/PD-L1 antibodies can block the activation of PD-1/PD-L1. APCs can absorb tumor antigens and regulate T-cell responses through interactions between the main MHC and TCR. APC can also regulate T-cell activity by regulating the interaction between PD-L1/PD-L2 and PD-1, as well as the interaction between B7 and CD28. The figure was generated by Biorender.com.

PD-1 is expressed on the surfaces of immune cells, such as T cells and B cells, with two ligands, PD-L1 (B7-H1, CD274) and PD-L2 (B7-DC, CD273) (159). PD-L1 is found on subsets of activated T cells, B cells, and macrophages, while PD-L2 is primarily on antigen-presenting cells (160). In physiological conditions, the PD-1/PD-L1 axis reduces inflammation-induced tissue damage and T-cell responses, playing a role in maintaining homeostasis to prevent autoimmune diseases (161). However, when activated T cells express PD-1 combined with tumor cells expressing PD-LI, the PD-1/PD-LI axis promotes immune escape from tumor cells through a variety of mechanisms (162). The PD-1/PD-LI axis induces T-cell tolerance by inhibiting TRC signaling (163). They can also inhibit the proliferation of CD8+ T lymphocytes to promote the death of antigen-specific T cells and tumor-infiltrating T lymphocytes, thereby suppressing the antitumor immune response (164). In addition, the PD-1/PD-LI axis can inhibit the PI3K/ALK and RAS/MEK/ERK signaling pathways to hinder the normal proliferation cycle of T lymphocytes, and the PD-1/PD-LI axis can downregulate the phosphorylation of mTOR, AKT, and ERK2 to upregulate the expression of PTEN, which promotes the transformation of CD4+ T cells into Tregs and inhibits the activity of effector T cells (165). An overview of PD-1/PD-L1 interaction-mediated T-cell inhibition is displayed in **Figure 2**. The immune checkpoint inhibitors used for the treatment of ovarian cancer in randomized clinical trials are summarized in **Table 2**.

**Table 2:** Immune checkpoint inhibitors used related agents for ovarian cancer in randomized clinical trials.

Checkpoint inhibitor	Clinicaltrials.gov	Status	Agents	Combination agents
	Identifier			
CTLA-4	NCT04678102	Recruiting	PHI-101	/
CTLA-4	NCT00094653	Completed	Ipilimumab	/
PD-1	NCT02674061	Completed	Pembrolizumab	Doxorubicin and Bevacizumab
	
	NCT02054806	Completed		
	
	NCT02865811	Completed		
PD-1	UMIN000005714	Unknown	Nivolumab	Ipilimumab
PD-L1	NCT01375842	Completed	Atezolizumab	Bevacizumab
PD-L1	NCT01772004	Completed	Avelumab	Axitinib, Docetaxel, and Doxolubicin
PD-L1	NCT02718417	Terminated	Avelumab	Carboplatin and Paclitaxel

Fukumoto et al. found that the combination of HDAC6 inhibition and anti-PD-L1 ICB exhibited a synergistic effect in OCs with ARID1A inactivation. This therapeutic strategy notably suppressed the transcription of CD274, the gene responsible for PD-L1 encoding. In preclinical mouse models treated with ACY1215, an HDAC6 inhibitor, and anti-PD-L1 ICB, there was a significant reduction in tumor burden and an improvement in survival rates. These favorable outcomes were linked to the activation and increase of IFNγ-positive CD8 T cells. These findings suggest that targeting HDAC6 in conjunction with ICB could represent a promising approach for treating cancers with ARID1A mutations (166). Shang et al. observed that transcript at the distal tip (HOTTIP), interleukin-6 (IL-6), and PD-L1 were all highly expressed in OC tissues. There was a positive correlation between IL-6 and PD-L1 levels; furthermore, HOTTIP was found to enhance IL-6 expression by binding to c-jun. This interaction subsequently promoted PD-L1 expression and facilitated immune escape in neutrophils, while inhibiting T cell proliferation and tumor immunotherapy effectiveness. The study suggests that HOTTIP’s role in increasing IL-6 secretion leads to enhanced PD-L1 expression in neutrophils, impairing T cell activity and promoting immune evasion by OC cells. Thus, targeting HOTTIP may offer a potential therapeutic strategy for OC (167). Cai et al. reported that PD-L1 was not significantly expressed in the TME of human OC. In contrast, B7-H3 was found to be highly expressed in both tumor cells and antigen-presenting cells within the tumor. This discrepancy highlights the complexity of immune responses in OC and suggests that other targets like B7-H3 may be crucial in developing effective therapies. Experiments conducted on mice with ID8 OvCa tumors revealed that B7-H3 expressed by tumor cells strongly inhibited the immune response against tumors, while its expression on host cells did not have the same effect. Blocking B7-H3 has been found to prolong survival in mice with ID8 tumors, indicating that B7-H3 expressed by tumors may inhibit the function of CD8+ T cells. This suggests that B7-H3 could be a potential therapeutic target for patients who do not respond effectively to PD-L1/PD-1 inhibitors (168). Lampert et al. observed varied responses among some OC patients, where a lower overall response rate (ORR) and a higher disease control rate (PRþSD) were reported. Treatment was associated with enhanced expression of several factors, increased systemic production of IFNγ and TNFα, and an increase in tumor-infiltrating lymphocytes, indicative of an immunostimulatory environment. Notably, higher IFNγ levels were linked to improved PFS, whereas elevated levels of VEGFR3 were associated with reduced PFS. These observations suggest that the combination of PARP inhibition (PARPi) and anti-PD-L1 therapy has modest clinical activity in recurrent OC. The use of olaparib/duvalizumab demonstrated an immunomodulatory effect in patients, highlighting the necessity of blocking the VEGF receptor pathway to enhance the efficacy of this combination treatment (169). Zhang et al. reported that the use of abcetinib as monotherapy significantly increased CD8+ T-cell and B-cell infiltration in an ID8 mouse model of OC, indicating potential benefits of this approach in enhancing anti-tumor immunity. The analysis showed that abercetinib induced proinflammatory immune responses in the TME. Compared with control cells, abciximab-treated ID8 cells secreted more CXCL10 and CXCL13. The synergistic antitumor effect of abemaciclib and anti-PD-1 combination therapy depended on CD8+ T cells and B cells. The research argues that the combination of cyclin-dependent kinases 4 and 6 inhibitors (CDK4/6i) with anti-PD-1 antibodies enhances the effectiveness of anti-PD-1 therapies and holds significant potential for the treatment of OC characterized by poor immune infiltration (170). Wan et al. identified that bivalent antibodies activate T cells and natural killer (NK) cells, leading to NK cells transitioning from an inactive to a more active cytotoxic state. This transformation suggests a substantial role of NK cells in the immune response induced by ICB in HGSC, highlighting the involvement of previously uncharacterized CD8 T cells in mounting an immune response. The observed alterations were partially attributed to the downregulation of the bromodomain-containing protein BRD1 by double-stranded antibodies. These findings suggest that modifying the state of NK cells and T cell subsets could be essential for eliciting an effective anti-tumor immune response and propose that immunotherapies, such as BRD1 inhibitors, which can induce such changes, may enhance treatment efficacy against HGSC (171). Yang et al. discovered that the presence of CXC-chemokine ligand 13 (CXCL13) in culture stimulates the expansion and activation of CXCR5+ CD8+ T cells *in vivo*. These T cells were found to be closer to CXCL13 within tumors and showed a propensity to migrate towards it *in vitro*. Additionally, patients with higher levels of CD20+ B cells and the presence of CXCL13 exhibited improved survival rates. When used in conjunction with anti-PD-1 therapy, CXCL13 effectively hindered tumor growth by enhancing the influx of cytotoxic CD8+ T cells and promoting the maintenance of CXCR5+ CD8+ T-cells within tertiary lymphoid structures (TLS). These findings strongly advocate for targeting CXCL13 along with PD-1 blockade as a potential therapeutic strategy for treating high-grade serous carcinoma (HGSC) (172). Laumont et al. found that the presence of CD39, CD103, and PD-1 together identifies specific subsets of TILs, namely CD8þ T cells and CD4þ regulatory Tregs. The co-expression of these markers correlates with a reduced diversity in the T cell receptor (TCR) among triple-positive CD8þ TILs. Additionally, triple-positive CD8þ effector cells uniquely showed higher expression of TIGIT compared to CD4þ Tregs. This simultaneous expression signifies the involvement of these highly activated immune cells in cytolytic, humoral, and regulatory immune responses. Notably, triple-positive TILs have significant prognostic value and present promising targets for combination immunotherapy involving PD-1 blockade as well as targeting both CD39 and TIGIT receptors (173). Seitz et al. found that overexpression of CXCL9 leads to T cell clustering, which results in delayed ascites formation and improved survival rates. This chemokine had effects similar to anti-PD-L1 therapy in an ICB-resistant mouse model, although the impacts were less pronounced in Brca2-deficient tumors. Moreover, the clear cell subtype, known for its high responsiveness to ICB among OC patients, exhibited a significantly greater prevalence of high CXCL9 tumors compared to other subtypes. These findings underscore the crucial role of CXCL9 in enhancing the efficacy of ICB treatments for preclinical OC and suggest that CXCL9 inducers could serve as both a viable predictive biomarker and a potent co-administration partner for improving ICB effectiveness in this cancer type (174). Dong et al. reported low expression of cullin 3 in OC (CUL3) and speckle-type POZ protein (SPOP). From a functional perspective, CUL3 degrades the PD-L1 protein by forming a complex with SPOP. By degrading the PD-L1 protein, CUL3 overexpression inhibited tumor formation, enhanced the chemosensitivity of mouse OC cells and reduced the malignant features and immune escape of OC cells. The findings indicate that the CUL3/SPOP complex facilitates PD-L1 degradation, thereby suppressing immune evasion and enhancing chemosensitivity in OC cells, presenting a promising therapeutic avenue for OC treatment (175). Lv-PD1-γδ T cells, developed by Wang et al., produced humanized anti-PD-1 antibodies. These cells amplified tumor cell proliferation and cytotoxicity, resulting in enhanced therapeutic efficacy and survival rates in mice harboring ovarian tumors. There was no potential tumorigenicity in immunocompromised NOD/SCID/γ-fasted mice. In addition, Lv-PD1- γδ T cells showed excellent tolerance and safety in humanized NOD/SCID/γ-fasted mice. The experimental results suggested that Lv-PD1-γδ T cells could attenuate or eliminate immunosuppression and maximize cytotoxicity by secreting anti-PD1 antibodies locally in the tumor and thus could serve as a promising anticancer “off-the-shelf” cell therapy (176).

#### 5.3.2 CTLA-4 inhibitors

The surface of T cells expresses CTLA-4, which is a molecule that acts as an immune checkpoint with inhibitory functions. CTLA-4 regulates T-cell activation and maintains immune system self-tolerance, preventing the immune system from attacking normal tissues (161). CTLA-4 competitively inhibits immune activation signaling and reduces T-cell activity by binding to B7 molecules (CD80 and CD86) (177). By blocking the function of CTLA-4, CTLA-4 inhibitors can inhibit T-cell activation, thereby improving the capacity of the immune system to recognize and attack cancer cells (178, 179). Although CTLA-4 inhibitors have shown significant effects in boosting immune responses, they may also lead to overactivation of the immune system to attack normal tissues and cause immune-related side effects (180). With an improved comprehension of immune checkpoint pathways and the advancement of novel immunotherapeutic approaches, the anticipation is that CTLA-4 inhibitors and their combined therapies will offer more efficacious treatment alternatives for a broader spectrum of cancer patients.

Friese et al. used a CTLA-4 blocking antibody, which was added during initial tumor-inflating lymphocyte (TIL) culture, and found that CTLA-4 blockade favored the propagation of CD8+ TILs in ovarian tumor fragments. In addition, the addition of CTLA-4 blocking antibody at the initial stage of TIL culture produced more potent antitumor TILs than did standard TIL culture. This phenotype was retained during the rapid amplification phase. These findings suggest that targeting CTLA-4 in the intact TME of a tumor fragment enriches tumor-responsive TILs and thus improves the clinical outcome of TIL-based ACT in OC (181). An investigation conducted by Swiderska et al. focused on three proteins associated with the immune response, namely, PD-1, PD-L1, and CTLA-4. To assess their effectiveness, receiver operating characteristic (ROC) curves were generated, and the area under the curve (AUC) was calculated to determine the sensitivity and specificity of these parameters. Utilizing Cox regression models, both univariate and multivariate analyses were carried out during this research endeavor. The findings from this study strongly suggest that considering CTLA-4 as a prospective biomarker could prove valuable in diagnosing OC while highlighting that elevated concentrations of PD-L1 and PD-1 serve as unfavorable prognostic indicators for this particular form of cancer (182). Chen et al. performed survival analyses on a subset of patients who were followed up. The results showed that the genotype and allele distribution frequency of the rs5742909 C/T polymorphism in cytotoxic T-lymphocyte antigen-4 (CTLA-4) differed significantly between patients and controls. Compared with the CC genotype, the CT + TT genotype significantly reduced the risk of developing EOC. However, no significant correlation was detected between the rs231775 G/A and rs3087243 G/A polymorphisms and susceptibility to EOC. The results confirmed that in women, the CTLA-4 rs5742909 C/T polymorphism may decrease the genetic predisposition to EOC (183).

#### 5.3.3 Tumor vaccines

Tumor vaccines are a new approach to immunotherapy and are considered a highly promising therapeutic modality in the field of tumor therapy. Tumor vaccines can induce body-specific antitumor immune responses by actively delivering tumor antigens to the body. Tumor vaccines can also make use of widely distributed T cells in the body to recognize and kill tumors (184). Tumor vaccines both improve the efficiency of tumor killing and overcome the drawbacks of conventional difficulties in completely removing tumors (161, 185). Currently, more clinical trials have been conducted in the field of tumor vaccines. However, most of the trials have small sample sizes, and a significant number of them have not achieved the expected results. Antigens, adjuvants, vectors, and the autoimmune status of the body play a dominant role in causing poor results in tumor vaccines. It is important to optimize the composition and timing of tumor vaccines with respect to these aspects to enhance the effectiveness of the vaccine. At the same time, the design of vaccines with individualized characteristics and preventive ability is highly important for improving tumor treatment and preventing tumor recurrence and metastasis.

Adams et al. demonstrated the potential of isolating ascites monocytes within the ID8 model and boosting their role as genuine antigen-presenting cells (APCs) by employing Toll-like receptor (TLR) 4 LPS, TLR9 CpG-oligonucleotide, and an interleukin-10 receptor (IL-10R) blocking antibody. Activated ascites monocytes efficiently curbed tumors and malignant ascites *in vivo*. Similarly, human ascites monocytes exhibited tumor-associated antigens (TAAs) under steady-state conditions. Notably, activated ascites mononuclear cells preserved their capacity to activate TALs even in the presence of ascites fluid. The findings suggest that ascites monocytes inherently harbor tumor antigens and can serve as potent antigen-presenting immune cells following a brief *in vivo* activation. This innovative ascites APC vaccine can be swiftly prepared, is straightforward, and cost-effective, making it an appealing option for OC treatment (186). Block et al. employed a Th17 induction protocol to generate DCs and loaded them with the HLA class II epitope of folate receptor alpha (FRα). Mature antigen-presenting DCs were subcutaneously injected. The majority of patients completing the respective interventions developed Th1, Th17, and FRα antibody responses post-vaccination. Prolonged relapse-free survival was associated with the presence of antibody-dependent cell-mediated cytotoxicity targeting FRα. Among the patients evaluable for efficacy, fewer than 40% remained relapse-free at the data cutoff. The findings indicate that vaccinating DCs with Th17-induced FRα loading is safe, induces antigen-specific immunity, and extends remission (187). Tanyi et al. improved vaccine-induced immune responses by combining ASA and low-dose IL-2 with the OCDC-Bev-Cy treatment. In the ID8 ovarian model, animals that received this treatment exhibited prolonged survival, elevated levels of perforin T cells within tumors and CD8+ T cells specific to neoantigens, and decreased expression of endothelial Fas ligand and intratumoral T-regulatory cells. These findings suggest that the ID8 model holds promise for the future development of OC trials (188). Nishida et al. observed a notable increase in the proportion of highly active tetrameric WT1-CTLs within CD8+ T lymphocytes (%tet-hi WT1-CTL) and a rise in WT1235-IgG levels post-vaccination. Furthermore, the elevated WT1235-IgG levels significantly prolonged progression-free survival. Unfavorable clinical outcomes associated with the WT1 vaccine included lower serum albumin levels, multiple tumor lesions, poor performance status, and excessive ascites. These results underscore that patients with refractory OC develop antigen-specific cellular and humoral immunity following WT1 vaccine administration. Both %tet-hi WT1-CTL and WT1235-IgG levels serve as prognostic markers for evaluating WT1 vaccine efficacy (189). Zhang et al. devised a fused cell membrane (FCM) nanovaccine, termed FCM-NPs. In addition, FCM-NPs exhibited the immunogenicity of tumor cells and the antigen-presenting ability of DCs and stimulated naïve T lymphocytes to produce large numbers of tumor-specific cytotoxic CD8+ T lymphocytes. FCM-NPs demonstrated robust immune activation both *in vitro* and *in vivo*. These findings highlight the potential of FCM-NPs to impede OC growth and hinder metastasis. FCM-NPs are poised to emerge as a novel tumor vaccine for OC treatment (190). As per Fucikova et al., although a higher tumor mutational burden (TMB) and significant CD8+ T-cell infiltration have been associated with improved outcomes in EOC patients undergoing conventional chemotherapy, this correlation does not apply to female patients undergoing DCVAC treatment. Conversely, positive responses to DCVAC were observed among individuals with below-average TMB levels and an excessive presence of CD8+ T-cells. These responses were accompanied by indications of enhanced effector function and specific tumor destruction within peripheral blood samples. In conclusion, our findings suggest that while heavily infiltrated “hot” EOC cases may benefit from chemotherapy, females diagnosed with less infiltrated “cold” EOC may require a dendritic cell-based vaccine as an alternative approach to elicit a clinically significant immune response against cancer (191). Zhao et al. conducted a study comparing the efficacy of co-formulated preparations of ICC and CPMV, either naturally bonded through CPMV-cell interactions or chemically coupled, with mixtures of PEGylated CPMV and ICC. The results revealed that blocking ICC interactions by PEGylation of CPMV did not significantly affect mice inoculated with the simple mixture of ICCs and (PEGylated) CPMV adjuvant. However, when co-formulated CPMV-ICCs were administered, approximately 70% of the mice survived, and 60% of the surviving mice successfully rejected tumor growth in a re-challenge experiment. These findings underscore the necessity of delivering cancer antigens and adjuvants together for effective OC vaccine development (192). Kos et al. reported a clinical therapeutic benefit in a small number of patients, including three partial responders (PR) and three patients with stable disease (SD) for more than six months. Clinical benefit was achieved in 1/3 of immunological responders and in less than 30% of evaluated patients. High levels of mRNA for various molecules linked to terminally differentiated T cells were expressed by immunological nonresponders in pretreatment peripheral blood mononuclear cell samples. These findings suggest that the combination of p53MVA and pembrolizumab immunotherapy shows promising efficacy against tumors in individuals with intact T-cell function in their peripheral blood. The identification of markers indicative of fully differentiated T cells prior to treatment could serve as a means to predict patients who are nonresponsive to p53MVA/pembrolizumab (193).

#### 5.3.4 Chimeric antigen receptor-T cells

**Figure 3:**
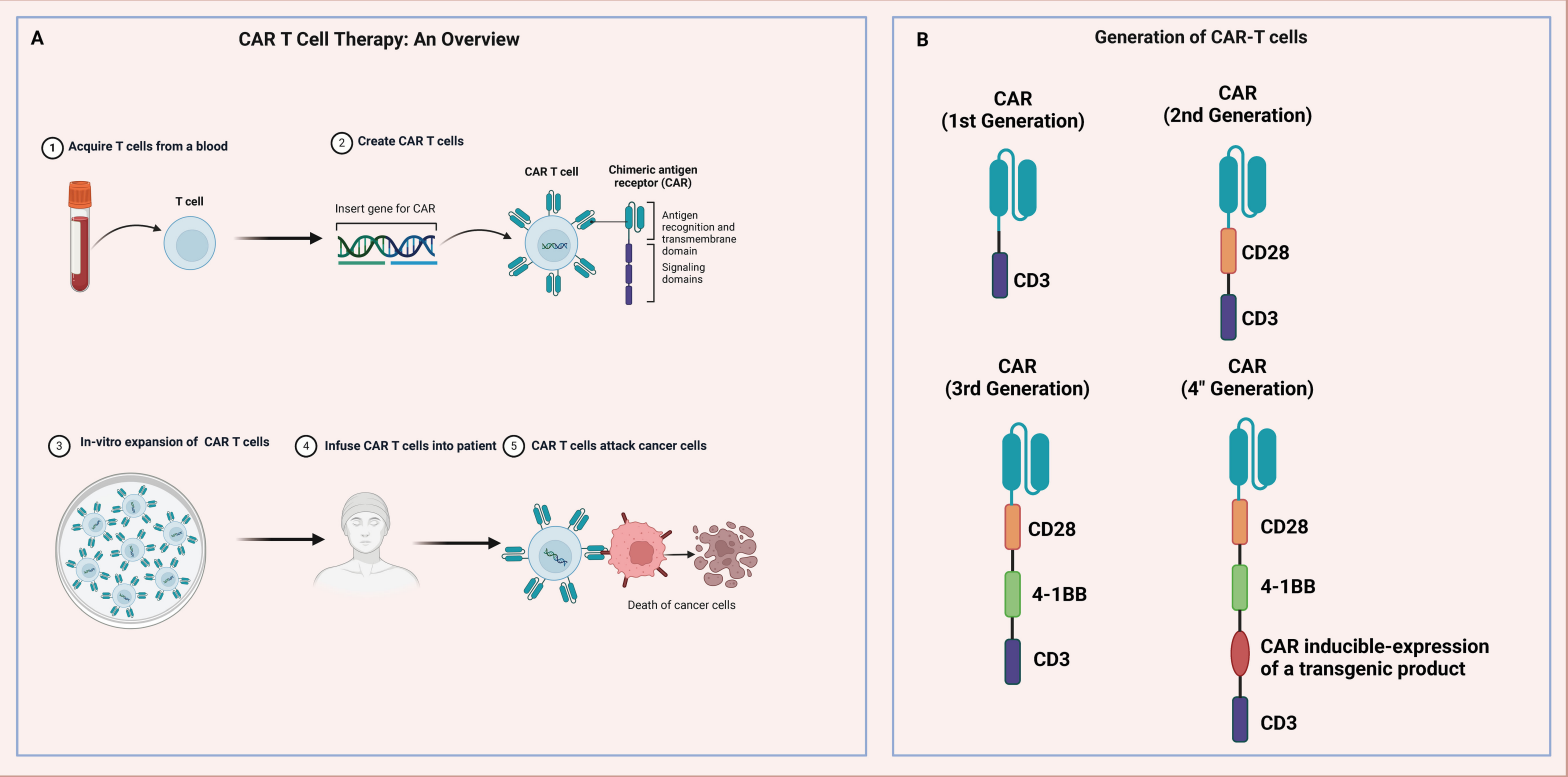
Generation of the brief and administration of CAR-T cells. The production and administration of CAR-T cells in cancer patients. **(A)** T cells are collected from patient blood through a single collection and genetically engineered to express CARs. After amplifying CAR-T cells *in vitro*, they are used in cancer patients. The reconstructed CAR-T cells are able to recognize their targets and kill tumor cells expressing that target. **(B)** Explanation of the basic structure of fourth-generation CAR-T cells. The figure was generated by Biorender.com.

CAR-T-cell immunotherapy is one of the more established forms of cellular therapy and involves the artificial modification of T cells taken from the patient’s body. T cells are artificially modified by the addition of chimeric antigen receptors (CARs), which can recognize tumor antigens, giving T cells the ability to recognize tumor cells. The modified T cells are then infused back into the body to achieve tumor recognition and killing (194). CAR-T-cell therapy was initially developed for certain types of hematological cancers (195) and has already achieved significant therapeutic results under these conditions. The successful use of CAR-T-cell therapy has stimulated widespread interest and research into its application in the treatment of solid tumors. The rapid generation and administration of CAR-T cells are shown in **Figure 3**, while the antigens used in CAR-T-cell therapy for ovarian cancer in randomized clinical trials are summarized in **Table 3**.

**Table 3:** Antigen used in CAR-T-cell therapy for ovarian cancer in randomized clinical trials.

Molecular	Clinicaltrials.gov	Status	Agents	Primary objectives
	Identifier			
Mesothelin	NCT03916679	Unknown	MSTL-CAR-T	The feasibility and safety of anti-MESO CAR-T cells in
	NCT03799913			treating patients with MESO-positive ovarian cancer.
	NCT04562298	Terminated	MSTL-CAR-T	Valuate the safety, tolerability, pharmacokinetics, and
				anti-tumor efficacy profiles of the LCAR-M23 CAR-T-cell therapy.
	NCT03814447	Unknown	MSTL-CAR-T	Determine the safety and feasibility of anti-MESO CAR-T
				cells therapy for Refractory-Relapsed Ovarian Cance
	NCT04503980	Unknown	MSTL-CAR-T	Valuate the safety and tolerability of autologous MSLN-CAR-T
				Cells secreting αPD1-MSLN-CAR T cells in patients with solid tumors.
	NCT05372692	Completed	MSTL-CAR-T	The effect of MSTL-CAR-T in patients with mesothelin-positive
				drug-resistant relapsed ovarian cancer.
	NCT01583686	Terminated	MSTL-CAR-T	Evaluate the safety of the administration of anti-mesothelin CAR engineered.
				peripheral blood lymphocytes in patients receiving a nonmyeloablative
				conditioning regimen, and aldesleukin. Determine if the administration
				anti-mesothelin CAR engineered peripheral blood lymphocytes and
				aldesleukin to patients following a nonmyeloablative but
				the lymphoid depleting preparative regimen will result in clinical
				tumor regression in patients with metastatic cancer.
	NCT03054298	Active, not recruiting	MSTL-CAR-T	Establish safety and feasibility of both intravenous administration and local
				delivery of lentiviral transduced huCART-meso cells with or
				without lymphodepletion.
	NCT02580747	Unknown	MSTL-CAR-T	Determine the safety and feasibility of the MESO CAR-T,
				determine the duration of *in vivo* survival of CART-meso cells.
	NCT05568680	Recruiting	MSTL-CAR-T	Assess the safety, feasibility, and potential activity of a single
				intravenous (IV) dose of SynKIR-110 administered to subjects with mesothelin-
				expressing advanced ovarian cancer, mesothelioma, and cholangiocarcinoma.
	NCT02159716	Completed	MSTL-CAR-T	Establish the safety and feasibility of intravenously administered lentiviral
				transduced CART-meso cells administered with and without cyclophosphamide
				in a 3 + 3 dose escalation design in patients with metastatic pancreatic cancer,
				serous epithelial ovarian cancer, or pleural mesothelioma.
	NCT03025256	Active, not recruiting	MSTL-CAR-T	To determine the safety and/or recommended dose of intrathecal (IT) nivolumab
				in combination with systemic nivolumab treatment in melanoma and
				lung cancer with leptomeningeal disease (LMD).
MUC16	NCT05239143	Recruiting	PD1-MUC16-CAR-T	To determine the safety, tolerability and response of P-MUC1C-ALLO1 in adult
				subjects with advanced or metastatic epithelial derived solid tumors.
MUC1	NCT04025216	Terminated	CART- TnMUC1	To determine the safety, tolerability, feasibility, and preliminary efficacy of
				CART-TnMUC1 cells engineered to express a CAR capable of recognizing
				the tumor antigen, TnMUC1 and activating the T cell.
TAG72	NCT05225363	Recruiting	TAG72-CAR T	To evaluate the safety and tolerability of TAG72-CAR T cells in
				participants with recurrent epithelial ovarian cancer (EOC).
				To determine the maximum tolerated dose (MTD). III. To identify the
				recommended phase 2 dose (RP2D).
FSHR	NCT05316129	Recruiting	FSHCER-CAR T	Evaluate the safety of treatment with FSHCER-CAR T, with or without
				conditioning chemotherapy in participants with recurrent or
				persistent ovarian, fallopian tube, or primary peritoneal cancer.

Garcia et al. reported that T cells expressing a specific chimeric antigen receptor (CAR) targeting the Müllerian inhibiting substance type 2 receptor (MISIIR) demonstrated targeted responses when exposed to antigens under laboratory conditions, effectively eradicating tumors with overexpressed MISIIR receptors within living organisms. Furthermore, these MISIIR CAR-T cells recognized various types of ovarian and endometrial cancer cell lines and exhibited the ability to destroy tumor samples obtained from patients without harming normal primary human cells. These results demonstrate that MISIIR-targeted therapy for OC and other gynecological malignancies can be achieved using CAR technology. *In vitro* experiments conducted by Li et al. illustrated the potent capacity of chimeric antigen receptor T (CAR-T) cells to eliminate OVCAR-3 cells while releasing a plethora of cytokines. Moreover, therapeutic efficacy was observed, along with significantly prolonged survival time in OVCAR-3 tumor-bearing mice. The experimental findings revealed that the killing capability of dual CAR-T cells on OVCAR-3 cells mirrored that of single CAR-T cells *in vitro*. However, *in vivo*, the killing efficacy of dual CAR-T cells led to a noteworthy enhancement and substantial extension of the lifespan of mice harboring tumors. *In vivo*, PD1-anti-MUC16 CAR-T cells exhibited stronger antitumor activity than single CAR-T cells. The current experimental data may lead to clinical studies (196). Shu et al. designed a truncated CD47 CAR without intracellular signaling structural domains. The CD47 CAR facilitates binding to CD47+ cells; thus, increasing the possibility of eradicating TAG72+ cells via the TAG72 CAR. In addition, we can reduce damage to normal tissues by monopolymerizing CD47 CAR. These findings suggest that expressing both TAG-72 CAR and CD47-truncated monoclonal CAR on T cells may be an effective dual CAR-T-cell strategy for the treatment of OC and is also applicable to other adenocarcinomas (197). Schoutrop et al. discovered that administering CAR T cells expressing an MSLN CAR construct incorporating the CD28 structural domain (M28z) led to a significant extension in survival. Despite a modest response rate, MSLN-4–1BB (MBBz) CAR T cells achieved long-term remission. Intratumorally infiltrated M28z and MBBz CAR T cells exhibited upregulation of PD-1 and LAG3 in response to antigens, while the respective ligands were expressed by MSLNþ-positive tumor cells. This suggests that immunosuppressive pathways within the ovarian tumor microenvironment hinder the persistence of CAR T cells. These findings highlight the promise of MSLN-CAR T cells for OC treatment (198). Guo et al. focused on the development of 5T4 CAR-T cells, which are second-generation human CAR-T cells engineered to specifically target the 5T4 protein. These CAR-T cells not only secrete cytotoxic cytokines but also exhibit lysogenic cytotoxicity against tumor cells. Additionally, the adoptive transfer of 5T4 CAR-T cells significantly delayed tumor formation. These findings underscore the potential effectiveness and viability of employing 5T4 CAR-T cell immunotherapy for OC treatment and lay a robust theoretical groundwork for future clinical studies targeting 5T4 cells (199). Liang et al. devised an innovative strategy to enhance the efficacy of CAR-T cell therapy by designing a tandem CAR that targets two antigens, one of which exhibits secretory activity (IL-12). *In vitro* experiments demonstrated that compared with single-target CAR-T cells, tandem CAR-T cells efficiently killed antigen-positive OC (OV) cells and showed enhanced secretion of cytokines. More importantly, the tandem CAR-T cells prolonged the survival of mice by reducing tumor size and enhancing antitumor activity. The results demonstrated that IL-12-secreting tandem CAR-T cells can enhance immunotherapeutic efficacy by reducing tumor antigen escape and improving T-cell function, which may be a promising strategy for the treatment of OV and other solid tumors (200). Chen et al. illustrated the effectiveness of anti-MSLN CAR-T cells in treating OC by inducing programmed cell death in MSLN-positive tumor cell lines. This led to suppressed tumor growth and increased cytokine levels compared to control groups. Subsequently, an *in vivo* experiment targeted OC-derived xenografts, demonstrating the safety and efficacy of autologous anti-MSLN CAR-T cell therapy for patients with this condition. These findings reinforce the potential efficacy of employing an anti-MSLN CAR-T treatment strategy for OC and offer initial data for future clinical trials (201). Shen et al. developed a novel CAR-T-cell therapy that effectively targets TM4SF1 for OC treatment. *In vitro* experiments demonstrated that CAR-T cells can specifically eliminate TM4SF1-positive tumor cell lines, while *in vivo* studies revealed that these modified immune cells significantly inhibited SKOV3-derived tumor growth. Possible therapeutic options for the management of OC may involve targeting TM4SF1, which has encouraging potential. Furthermore, there is a possibility for the advancement of immunotherapy utilizing TM4SF1 as a basis (202). Schoutrop et al. evaluated the therapeutic efficacy of MSLN-CAR-T cells and the characteristics and number of different MSLN-CAR-T cells and found that M1xx CAR-T cells had greater antitumor potency and durability than conventional second-generation M28z and MBBz CAR-T cells. In addition, M1xx CAR-T cells exhibited improved *in vitro* generation capacity and were characterized by self-renewal genes. These findings indicate that MSLN-CAR T cells, which express a mutated CD3ζ strand containing only one immunoreceptor tyrosine-based activation motif (ITAM), demonstrate enhanced potential for treating OC. Employing CAR T cells with precisely calibrated activation potential may lead to improved clinical responses in solid tumors (203). Ranoa et al. conducted a comparative analysis on the efficacy of four Tn-dependent CARs with different affinities toward the Tn antigen. The 237 CAR and a mutant with significantly higher affinity, along with a CAR with lower affinity, effectively controlled advanced ID8Cosmc-KO tumors. Tumor regression was more pronounced with a single dose of intraperitoneal intravenous CAR. The most successful CARs were associated with the antigen possessing the highest affinity. Furthermore, less effective CARs exhibited tonic signaling, leading to cytokine expression independent of the antigen. These findings provide evidence for the potential use of affinity-enhanced CAR-T cells in treating advanced OC, where different patterns of inflammatory cytokine release were observed *in vitro* among successful CARs. Importantly, Tn-dependent CAR-T cells, which are considered highly effective, do not exhibit any toxicity to the immune system of tumor-bearing mice (204). Xu et al. conducted a thorough investigation into the therapeutic potential of PTK7 CAR-T cells against OC, both *in vitro* and *in vivo*. Their study unveiled significant PTK7 expression in OC tissues and cells, indicating its suitability as a target for CAR-T cell therapy. Through the TREM1/DAP12 signaling pathway, researchers observed robust cytotoxicity of PTK7-targeted CAR-T cells against OC cells expressing PTK7 in laboratory settings. Additionally, these engineered immune cells effectively eradicated tumors in animal models. These findings suggest that employing TREM1/DAP12-based PTK7 CAR-T cell therapy shows promise as an innovative approach for treating OC; however, further evaluation is necessary to determine its safety profile and clinical efficacy through rigorous clinical trials (205). Mun et al. developed Muc16-specific CAR T cells (4H11) capable of producing a bispecific T cell engager (BiTE), composed of a TCR mimetic antibody (ESK1). This BiTE selectively binds to the WT1-derived epitope RMFP, presented by the HLA-A2 molecule. Compared with 4H11 CAR-T cells alone, secreted ESK1 BiTE redirected additional T cells toward WT1 on tumor cells, resulting in improved anticancer activity against Muc16-overexpressing cancer cells. These observations were made in both laboratory experiments and a mouse model of tumors. This novel strategy of dual orthogonal cytotoxicity targets distinct surface and intracellular tumor-associated antigens, indicating potential for overcoming resistance to CAR-T cell therapies not only in EOC but also in other malignancies (206). Mondal et al. characterized an important cluster of positively charged residues known as PPCR within domain 2 of the Fas protein structure. This cluster plays a crucial role in blocking apoptotic signaling triggered by mutated forms of either FasL or Fas, thereby affecting both tumor cells and T cells. Furthermore, our study sheds light on how Fasl interacts with its receptor, Fas, at the PPCR interface through various mechanisms. These findings suggest that employing death agonists could potentially offer an effective therapeutic avenue for targeting FAS and improving cancer immunotherapy outcomes, not only for OC but also for various solid tumors (207). Biotec et al. verified the high expression and consistency of the tumor-associated antigen FOLR1 in primary OC samples. Subsequently, a range of potential CAR T cells were engineered to target the identified markers, and their efficacy against OC cell lines was validated in laboratory and animal models. Ultimately, an automated manufacturing process for these candidate CAR T cells was developed through additional laboratory tests. These results underscore the potential of utilizing anti-FOLR1 CAR T cells as a therapeutic strategy for treating OC and other tumors expressing FOLR1 (208).

## 6 Discussion and prospects

**Figure 4:**
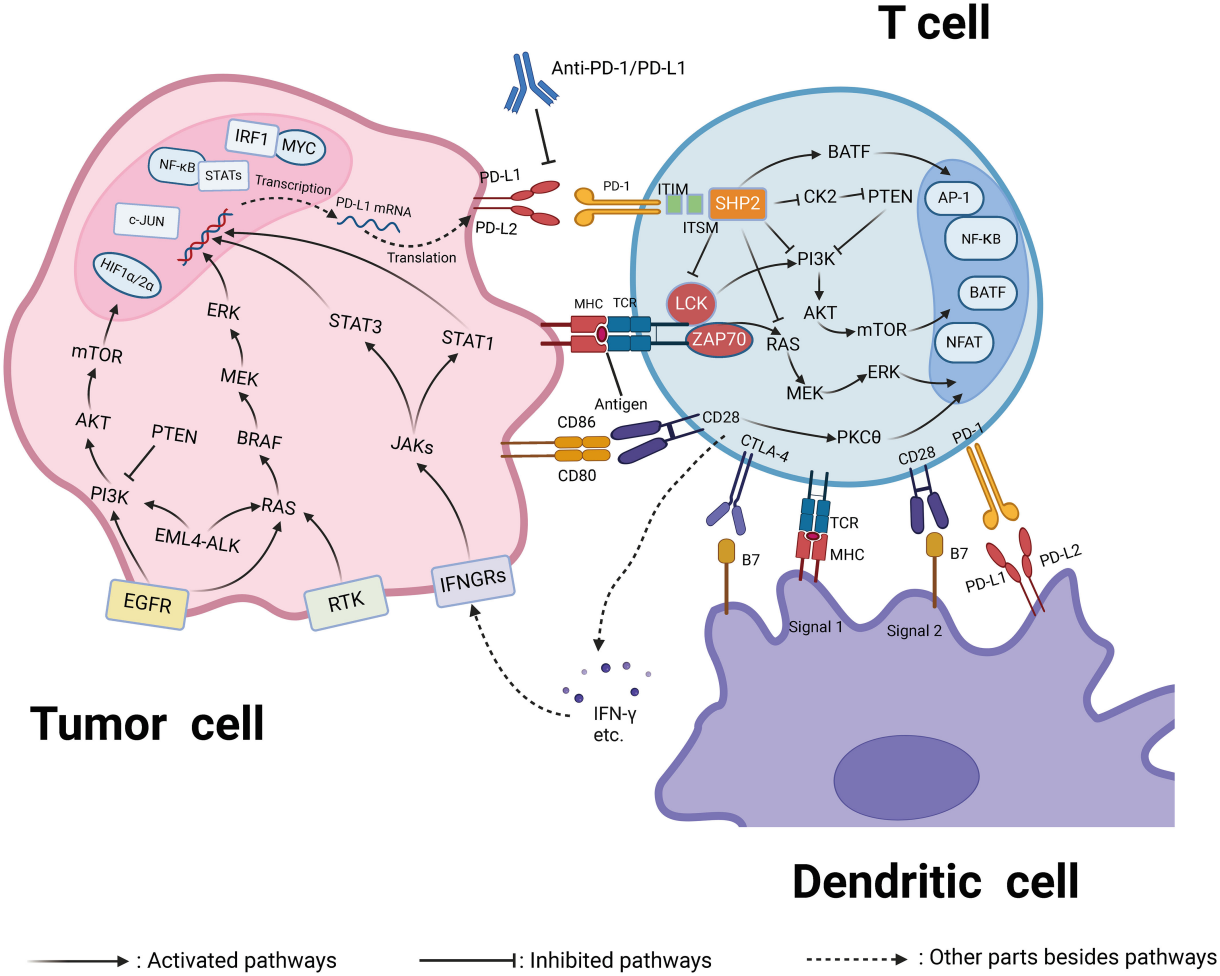
The components and therapeutic targets of the tumor microenvironment (TME). The TME is composed of various cells, including cancer stem cells, immune cells, and stromal cells. Treatment targets and intervention measures for the TME. Drugs approved by the US Food and Drug Administration for targeting VEGF, CTLA-4, EGFR, PD-L1, and PD-1.

OC is difficult to diagnose at an early stage, and its five-year survival rate is low. Henceforth, it is crucial to develop new methods for early screening of OC and new therapeutic strategies. As the role of the TME in OC metastasis has received increasing attention, understanding the interactions between immune cell molecules in the TME and tumor cells at primary and metastatic sites and elucidating the molecular mechanisms of the TIME and tumor cells in OC metastasis are urgently needed to provide guidance for the clinical development of new diagnostic markers and therapeutic targets for OC. Since most tumor cells are weakly immunogenic, it is difficult to induce specific immune responses against these antigens in the body. Immunotherapies, such as vaccines to stimulate the host antitumor immune response, the application of genetically engineered cytokines, the infusion of immune effector cells, etc., to assist surgery, radiotherapy, and chemotherapy, in the treatment of OC, have promising application prospects. The intricate interplay between cancer genomic alterations and the complex microenvironment poses challenges to the development of effective immunotherapies. Successful immunotherapy for OC hinges on stimulating antigen-presenting cells, mitigating the suppressive immune microenvironment, and enhancing effector T-cell activity. The modulation of cell-mediated immune responses entails both inhibitory and stimulatory signals. Immune checkpoint receptors play a crucial role in dampening T-cell activation to prevent hyperactivation. **Figure 4** illustrates the constituents and therapeutic targets of the TME.

Certain types of tumors express immune checkpoints, allowing them to evade the immune system hence blocking these checkpoints is crucial in immunotherapy. Lysovirus therapy, a novel anti-tumor strategy, triggers an immune response against tumors by rapidly proliferating and ultimately killing tumor cells in response to lysovirus infection (209). While immune checkpoint inhibitors have shown promise in various tumors, their efficacy in OC therapy is relatively low. To enhance the anti-tumor response rate, several studies are combining them with chemotherapy or small molecule inhibitors as adjuvant therapy. However, applying CAR-T cell therapy to solid tumors faces challenges like effective penetration into tumor tissues, overcoming immunosuppressive factors, and minimizing damage to normal tissues. Despite these challenges, CAR-T cell therapy holds significant potential in cancer treatment. Ongoing research explores new target antigens, improved CAR designs, novel management strategies, and combination therapies to expand CAR-T therapy’s application and efficacy. As research advances, CAR-T cell therapy is expected to offer hope to more cancer patients. Immunotherapy represents a paradigm shift in OC treatment, but effectively enhancing its efficacy remains a priority. A deeper understanding of the OC immune microenvironment and immune escape mechanisms could lead to novel therapeutic strategies. By using cutting-edge technologies, personalized immunotherapy can be implemented by considering tumor biology and the microenvironment. Therefore, investigating the integration of chemotherapy with immunotherapy or other strategies is essential to improve efficacy and mitigate immune escape in OC treatment.

Given its poor prognosis and high mortality rates, OC has presented significant challenges in treatment, especially in advanced stages where options are scarce. Immunotherapy holds potential for OC treatment, offering better survival outcomes, long-lasting responses, and manageable safety profiles in advanced cases. Nonetheless, current single-agent studies have not yielded substantial survival benefits. In addition to the immunosuppressive properties of OC, this may be due to the complexity of the OC microenvironment, which prevents the activity of immune cells and should, therefore, be targeted by the TME. Temporal factors play a crucial role in modulating the suppression of the immune response and tolerance, as well as influencing the quantity and functionality of TILs. Elucidating the interplay between OC cells and the surrounding mesenchymal environment is imperative for identifying effective therapeutic approaches and reliable prognostic biomarkers. All elements of the TME must be taken into account when devising new therapeutic approaches, particularly for “variable tumors” or “cold tumors.” These strategies may involve initiating T-cell responses, utilizing vaccines and CAR-T cells targeting neoantigens in tumor cells, inducing immunogenic cell death in *in situ* vaccination strategies, and simultaneously targeting checkpoint inhibitors responsible for T-cell dysfunction or failure.

## 7 Conclusion and prospectives

In this study, we conducted a thorough analysis of the diverse cellular components within the TME and their functions in controlling OC progression and treatment responses. Furthermore, we offered a detailed evaluation of the obstacles linked to ICI-based therapies in OC. Altogether, advancing our understanding of the TME will facilitate the development of precise and effective treatment approaches for OC.

The tools currently employed to examine the TME include genomics to investigate gene expression features in high-grade serous ovarian cancer. Verhaak et al. identified four distinct genetic categories in a study on ovarian tumors, designated as differentiated, immune responsive, mesenchymal, and proliferative (210). It has been determined through IHC that T lymphocytes are increased in the immune-responsive group, while an increase in connective tissue proliferation associated with infiltrating stromal cells is noted in the mesenchymal group. The survival outcomes for patients in the immune-responsive group are the most favorable. Several malignant tissues exhibit more than one of these four gene clusters. These findings were corroborated using an independent dataset of 879 high-grade serous ovarian cancer expression profiles. Additional insights into survival outcomes and platinum resistance were gained using survival prediction models related to BRCA1/2 mutation status, postoperative residual disease, and disease staging (210). Therefore, the development of similar gene classification models might assist in selecting patients for targeted or immunotherapy, or in predicting patient prognosis. For instance, it has been observed that patients exhibiting mesenchymal traits may respond more effectively to therapies such as angiogenic inhibitors. Additional methods for studying the high-grade serous ovarian carcinoma TME include integrating proteomics with other genomic data (211), and employing multiple parameter analysis techniques (such as gene expression, matrix proteomics, cytokine and chemokine expression, ECM parameters, and biomechanical properties) on single biopsy samples to enhance understanding of the events occurring within the tumor tissue (212). Novel tools for investigating ovarian TME include the utilization of artificial microenvironments to monitor the progression of ovarian cancer (213). The TME is a complex and dynamic entity that may vary between primary disease and recurrence. In devising more effective treatments, it is crucial to consider existing immune suppression and emerging mechanisms of therapeutic resistance (214). Cancer treatment with immunotherapy has not achieved the same success as treatments for other types of cancer (215). Combining immunotherapy, such as PD-1 blockade, with other checkpoint inhibitory molecules like anti CTLA-4, anti TIM-3, anti LAG-3, PARP inhibitors, kinase inhibitors, chemotherapy drugs (216), dendritic cell vaccines, CAR T cell therapy (217), or other therapies have been validated to be successful measures to overcome multiple immunosuppressive mechanisms in TME. In fact, targeting complements in OC-TME is a new approach to developing effective immunotherapy. A randomized phase 2 clinical trial (NCT04919629) is currently underway, investigating the combination of a complement inhibitor, anti-PD1, and anti-VEGF for recurrent ovarian cancer patients. The results of this trial may guide future strategies involving complement inhibition in ovarian cancer TME alongside other treatments.

## Author contributions

JC: Writing – review & editing, Writing – original draft. LY: Writing – original draft, Validation. YM: Writing – review & editing, Visualization, Investigation. YZ: Writing – review & editing, Writing – original draft, Visualization, Validation.

## Glossary

**Table:**

OC	ovarian cancer
EOC	epithelial ovarian cancer
TME	tumor microenvironment
ICs	immune checkpoints
ICIs	immune checkpoint inhibitors
ECM	extracellular matrix
CAFs	cancer-associated fibroblasts
TACs	tumor-associated cells
TAMs	tumor-associated macrophages
NK	natural killer
DCs	dendritic cells
TANs	tumor-associated neutrophils
MDSCs	myeloid-derived suppressor cells
CTLs	cytotoxic T lymphocytes
CTL	cytotoxic T lymphocytes
NK	natural killer
PDK1	pyruvate dehydrogenase kinase 1
VEGF	vascular endothelial growth factor
TGF-β	tumor growth factor-β
CTLA-4	cytotoxic T-lymphocyte antigen-4
IL-10R Ab	interleukin-10 receptor-blocking antibody
TLR	Toll-like receptor
LPS	lipopolysaccharide
rCTHRC1	recombinant CTHRC1 protein
CRIg	complement receptor
CCC	clear cell carcinoma
DDP	TPL+cisplatin
ZEB1	zinc E box binding isozyme 1
s mCAFs	metastatic CAFs
POSTN	periostin
CM	conditioned media
DDR2	Discoidin Domain Receptor 2
NFs	normal fibroblasts
EVs	extracellular vesicles
OS	overall survival
cKO	stroma-specific knockout
NFs	normal fibroblasts
EMT	epithelial-mesenchymal transition
CDDP	cisplatin
CRMP2	Collapsin response mediator protein-2
SDF-1α	stromal derived factors-1α
TIME	tumor immune microenvironments
PF	peritoneal fluid
CSF2	colon-stimulating factor 2
PARPi	PARP inhibition
ORR	overall response rate
APCs	antigen-presenting cells
CXCL13	CXC- chemokine ligand 13
ICB	immune checkpoint blockade
TIL	tumor-inflating lymphocyte
TAA	tumor-associated antigen
TLR	Toll-like receptor
FCM	fused cell membrane
CAR	chimeric antigen receptor
CAR-T	chimeric antigen receptor T
RMF	RMFPNAPYL
ALPS	autoimmune lymphoproliferative syndrome

## References

[1] St Laurent J Liu JF. Treatment approaches for platinum-resistant ovarian cancer. J Clin Oncol. (2024) 42:127–33. doi: 10.1200/JCO.23.01771 37910841

[2] Wang L Wang X Zhu X Zhong L Jiang Q Wang Y. Drug resistance in ovarian cancer: from mechanism to clinical trial. Mol Cancer. (2024) 23:66. doi: 10.1186/s12943-024-01967-3 38539161 PMC10976737

[3] Ostrowska-Lesko M Rajtak A Moreno-Bueno G Bobinski M. Scientific and clinical relevance of non-cellular tumor microenvironment components in ovarian cancer chemotherapy resistance. Biochim Biophys Acta Rev Cancer. (2024) 1879:189036. doi: 10.1016/j.bbcan.2023.189036 38042260

[4] Tadić V Zhang W Brozovic A. The high-grade serous ovarian cancer metastasis and chemoresistance in 3D models. Biochim Biophys Acta Rev Cancer. (2024) 1879:189052. doi: 10.1016/j.bbcan.2023.189052 38097143

[5] Guerra C Kalaitsidou M Kueberuwa G Hawkins R Edmondson R. Engineering strategies to optimise adoptive cell therapy in ovarian cancer. Cancer Treat Rev. (2023) 121:102632. doi: 10.1016/j.ctrv.2023.102632 37837788

[6] Schettini F Venturini S Giuliano M Lambertini M Pinato DJ Onesti CE. Multiple Bayesian network meta-analyses to establish therapeutic algorithms for metastatic triple negative breast cancer. Cancer Treat Rev. (2022) 111:102468. doi: 10.1016/j.ctrv.2022.102468 36202026

[7] Wang Y Duval AJ Adli M Matei D. Biology-driven therapy advances in high-grade serous ovarian cancer. J Clin Invest. (2024) 134:e174013. doi: 10.1172/JCI174013 38165032 PMC10760962

[8] Carlino MS Larkin J Long GV. Immune checkpoint inhibitors in melanoma. Lancet. (2021) 398:1002–14. doi: 10.1016/S0140-6736(21)01206-X 34509219

[9] Wang A Wang Y Du C Yang H Wang Z Jin C. Pyroptosis and the tumor immune microenvironment: A new battlefield in ovarian cancer treatment. Biochim Biophys Acta Rev Cancer. (2023) 1879:189058. doi: 10.1016/j.bbcan.2023.189058 38113952

[10] Bahadoer RR Dijkstra EA van Etten B Marijnen CAM Putter H Kranenbarg EM. Short-course radiotherapy followed by chemotherapy before total mesorectal excision (TME) versus preoperative chemoradiotherapy, TME, and optional adjuvant chemotherapy in locally advanced rectal cancer (RAPIDO): a randomised, open-label, phase 3 trial. Lancet Oncol. (2021) 22:29–42. doi: 10.1016/S1470-2045(20)30555-6 33301740

[11] Yu P Wang Y Yuan D Sun Y Qin S Li T. Vascular normalization: reshaping the tumor microenvironment and augmenting antitumor immunity for ovarian cancer. Front Immunol. (2023) 14:1276694. doi: 10.3389/fimmu.2023.1276694 37936692 PMC10626545

[12] Hanahan D Weinberg RA. Hallmarks of cancer: the next generation. Cell. (2011) 144:646–74. doi: 10.1016/j.cell.2011.02.013 21376230

[13] Chen L Flies DB. Molecular mechanisms of T-cell costimulation and coinhibition. Nat Rev Immunol. (2013) 13:227–42. doi: 10.1038/nri3405 PMC3786574 23470321

[14] Gong J Chehrazi-Raffle A Reddi S Salgia R. Development of PD-1 and PD-L1 inhibitors as a form of cancer immunotherapy: a comprehensive review of registration trials and future considerations. J Immunother Cancer. (2018) 6:8. doi: 10.1186/s40425-018-0316-z 29357948 PMC5778665

[15] Kurtz JE Pujade-Lauraine E Oaknin A Belin L Tsibulak I Cibula D. LBA30 Phase III ATALANTE/ov29 trial: Atezolizumab (Atz) versus placebo with platinum-based chemotherapy (Cx) plus bevacizumab (bev) in patients (pts) with platinum-sensitive relapse (PSR) of epithelial ovarian cancer (OC). Ann Oncol. (2022) 33:S1397. doi: 10.1016/j.annonc.2022.08.026

[16] Monk BJ Colombo N Oza AM Fujiwara K Birrer MJ Randall L. Chemotherapy with avelumab or other drug followed by avelumab maintenance versus chemotherapy alone in patients with previously untreated epithelial ovarian cancer (JAVELIN Ovarian 100): an open-label, randomised, phase 3 trial. Lancet Oncol. (2021) 22:1275–89. doi: 10.1016/S1470-2045(21)00342-9 34363762

[17] Chen S Xu Y Zhuo W Zhang L. The emerging role of lactate in tumor microenvironment and its clinical relevance. Cancer Lett. (2024) 590:216837. doi: 10.1016/j.canlet.2024.216837 38548215

[18] Wang Z Sun W Hua R Wang Y Li Y Zhang H. Promising dawn in tumor microenvironment therapy: engineering oral bacteria. Int J Oral Sci. (2024) 16:24. doi: 10.1038/s41368-024-00282-3 38472176 PMC10933493

[19] Khan C Rusan NM. Using Drosophila to uncover the role of organismal physiology and the tumor microenvironment in cancer. Trends Cancer. (2024) 10:289–311. doi: 10.1016/j.trecan.2024.01.007 PMC11008779 38350736

[20] Lin J Rao D Zhang M Gao Q. Metabolic reprogramming in the tumor microenvironment of liver cancer. J Hematol Oncol. (2024) 17:6. doi: 10.1186/s13045-024-01527-8 38297372 PMC10832230

[21] Cerchietti L. Genetic mechanisms underlying tumor microenvironment composition and function in diffuse large B-cell lymphoma. Blood. (2024) 143:1101–11. doi: 10.1182/blood.2023021002 PMC10972714 38211334

[22] Berrell N Sadeghirad H Blick T Bidgood C Leggatt GR O'Byrne K. Metabolomics at the tumor microenvironment interface: Decoding cellular conversations. Medicinal Res Rev. (2023) 44:1121–1146. doi: 10.1002/med.22010 38146814

[23] Wang X Luo Y Ma Y Wang P Yao R. Converging bioprinting and organoids to better recapitulate the tumor microenvironment. Trends Biotechnol. (2023) 42:648–663. doi: 10.1016/j.tibtech.2023.11.006 38071145

[24] Walsh LA Quail DF. Decoding the tumor microenvironment with spatial technologies. Nat Immunol. (2023) 24:1982–93. doi: 10.1038/s41590-023-01678-9 38012408

[25] Lin Q Choyke PL Sato N. Visualizing vasculature and its response to therapy in the tumor microenvironment. Theranostics. (2023) 13:5223–46. doi: 10.7150/thno.84947 PMC10614675 37908739

[26] Li Z Yin P. Tumor microenvironment diversity and plasticity in cancer multidrug resistance. Biochim Biophys Acta Rev Cancer. (2023) 1878:188997. doi: 10.1016/j.bbcan.2023.188997 37832894

[27] Zheng Y Sun L Guo J Ma J. The crosstalk between ferroptosis and anti-tumor immunity in the tumor microenvironment: molecular mechanisms and therapeutic controversy. Cancer Commun (London England). (2023) 43:1071–96. doi: 10.1002/cac2.12487 PMC10565387 37718480

[28] Huang Q Zhong X Li J Hu R Yi J Sun J. Exosomal ncRNAs: Multifunctional contributors to the immunosuppressive tumor microenvironment of hepatocellular carcinoma. BioMed Pharmacother. (2024) 173:116409. doi: 10.1016/j.biopha.2024.116409 38460375

[29] Li D Cao D Sun Y Cui Y Zhang Y Jiang J. The roles of epigallocatechin gallate in the tumor microenvironment, metabolic reprogramming, and immunotherapy. Front Immunol. (2024) 15:1331641. doi: 10.3389/fimmu.2024.1331641 38348027 PMC10859531

[30] Al-Bzour NN Al-Bzour AN Ababneh OE Al-Jezawi MM Saeed A Saeed A. Cancer-associated fibroblasts in gastrointestinal cancers: unveiling their dynamic roles in the tumor microenvironment. Int J Mol Sci. (2023) 24:16505. doi: 10.3390/ijms242216505 38003695 PMC10671196

[31] Zhang W Wang J Liu C Li Y Sun C Wu J. Crosstalk and plasticity driving between cancer-associated fibroblasts and tumor microenvironment: significance of breast cancer metastasis. J Trans Med. (2023) 21:827. doi: 10.1186/s12967-023-04714-2 PMC10657029 37978384

[32] Zhang Z Chen X Gao S Fang X Ren S. 3D bioprinted tumor model: a prompt and convenient platform for overcoming immunotherapy resistance by recapitulating the tumor microenvironment. Cell Oncol (Dordrecht). (2024). doi: 10.1007/s13402-024-00935-9 PMC11322267 38520648

[33] Saad EE Michel R Borahay MA. Immunosuppressive tumor microenvironment and uterine fibroids: Role in collagen synthesis. Cytokine Growth factor Rev. (2024) 75:93–100. doi: 10.1016/j.cytogfr.2023.10.002 37839993 PMC10922281

[34] Kay EJ Zanivan S Rufini A. Proline metabolism shapes the tumor microenvironment: from collagen deposition to immune evasion. Curr Opin Biotechnol. (2023) 84:103011. doi: 10.1016/j.copbio.2023.103011 37864905

[35] Zhang H Yue X Chen Z Liu C Wu W Zhang N. Define cancer-associated fibroblasts (CAFs) in the tumor microenvironment: new opportunities in cancer immunotherapy and advances in clinical trials. Mol Cancer. (2023) 22:159. doi: 10.1186/s12943-023-01860-5 37784082 PMC10544417

[36] Xu Y Li W Lin S Liu B Wu P Li L. Fibroblast diversity and plasticity in the tumor microenvironment: roles in immunity and relevant therapies. Cell communication signaling: CCS. (2023) 21:234. doi: 10.1186/s12964-023-01204-2 37723510 PMC10506315

[37] Wu J Ji H Li T Guo H Xu H Zhu J. Targeting the prostate tumor microenvironment by plant-derived natural products. Cell signalling. (2024) 115:111011. doi: 10.1016/j.cellsig.2023.111011 38104704

[38] Yang J Bae H. Drug conjugates for targeting regulatory T cells in the tumor microenvironment: guided missiles for cancer treatment. Exp Mol Med. (2023) 55:1996–2004. doi: 10.1038/s12276-023-01080-3 37653036 PMC10545761

[39] Pan Y Yang W Tang B Wang X Zhang Q Li W. The protective and pathogenic role of Th17 cell plasticity and function in the tumor microenvironment. Front Immunol. (2023) 14:1192303. doi: 10.3389/fimmu.2023.1192303 37457739 PMC10339829

[40] Ji S Shi Y Yin B. Macrophage barrier in the tumor microenvironment and potential clinical applications. Cell communication signaling: CCS. (2024) 22:74. doi: 10.1186/s12964-023-01424-6 38279145 PMC10811890

[41] Qian Y Yin Y Zheng X Liu Z Wang X. Metabolic regulation of tumor-associated macrophage heterogeneity: insights into the tumor microenvironment and immunotherapeutic opportunities. biomark Res. (2024) 12:1. doi: 10.1186/s40364-023-00549-7 38185636 PMC10773124

[42] Tang BF Yan RC Wang SW Zeng ZC Du SS. Maternal embryonic leucine zipper kinase in tumor cells and tumor microenvironment: An emerging player and promising therapeutic opportunity. Cancer Lett. (2023) 560:216126. doi: 10.1016/j.canlet.2023.216126 36933780

[43] Li L Tian Y. The role of metabolic reprogramming of tumor-associated macrophages in shaping the immunosuppressive tumor microenvironment. BioMed Pharmacother. (2023) 161:114504. doi: 10.1016/j.biopha.2023.114504 37002579

[44] Moinuddin A Poznanski SM Portillo AL Monteiro JK Ashkar AA. Metabolic adaptations determine whether natural killer cells fail or thrive within the tumor microenvironment. Immunol Rev. (2024) 323:19–39. doi: 10.1111/imr.13316 38459782

[45] Miao L Lu C Zhang B Li H Zhao X Chen H. Advances in metabolic reprogramming of NK cells in the tumor microenvironment on the impact of NK therapy. J Trans Med. (2024) 22:229. doi: 10.1186/s12967-024-05033-w PMC10909296 38433193

[46] Hu T Shi R Gu Y Zhou H Fang Y Xu T. Cancer-derived non-coding RNAs endow tumor microenvironment with immunosuppressive properties. Wiley Interdiscip Rev RNA. (2023) 10:e1822. doi: 10.1002/wrna.1822 37817381

[47] Xiao Z Wang R Wang X Yang H Dong J He X. Impaired function of dendritic cells within the tumor microenvironment. Front Immunol. (2023) 14:1213629. doi: 10.3389/fimmu.2023.1213629 37441069 PMC10333501

[48] Tian X Wang T Shen H Wang S. Tumor microenvironment, histone modifications, and myeloid-derived suppressor cells. Cytokine Growth factor Rev. (2023) 74:108–21. doi: 10.1016/j.cytogfr.2023.08.002 37598011

[49] Budi HS Farhood B. Targeting oral tumor microenvironment for effective therapy. Cancer Cell Int. (2023) 23:101. doi: 10.1186/s12935-023-02943-5 37221555 PMC10207626

[50] Wu M Zhou S. Harnessing tumor immunogenomics: Tumor neoantigens in ovarian cancer and beyond. Biochim Biophys Acta Rev Cancer. (2023) 1878:189017. doi: 10.1016/j.bbcan.2023.189017 37935309

[51] Konstantinopoulos PA Matulonis UA. Clinical and translational advances in ovarian cancer therapy. Nat Cancer. (2023) 4:1239–57. doi: 10.1038/s43018-023-00617-9 37653142

[52] Gao Q Yang Z Xu S Li X Yang X Jin P. Heterotypic CAF-tumor spheroids promote early peritoneal metastatis of ovarian cancer. J Exp Med. (2019) 216:688–703. doi: 10.1084/jem.20180765 30710055 PMC6400537

[53] Kandalaft LE Dangaj Laniti D Coukos G. Immunobiology of high-grade serous ovarian cancer: lessons for clinical translation. Nat Rev Cancer. (2022) 22:640–56. doi: 10.1038/s41568-022-00503-z 36109621

[54] Barua A Bahr JM. Ovarian cancer: applications of chickens to humans. Annu Rev Anim Biosci. (2022) 10:241–57. doi: 10.1146/annurev-animal-021419-084001 35167319

[55] Cheng Y Song S Wu P Lyu B Qin M Sun Y. Tumor associated macrophages and TAMs-based anti-tumor nanomedicines. Advanced healthcare materials. (2021) 10:e2100590. doi: 10.1002/adhm.202100590 34292673

[56] An D Banerjee S Lee JM. Recent advancements of antiangiogenic combination therapies in ovarian cancer. Cancer Treat Rev. (2021) 98:102224. doi: 10.1016/j.ctrv.2021.102224 34051628 PMC8217312

[57] El Bairi K Al Jarroudi O Afqir S. Revisiting antibody-drug conjugates and their predictive biomarkers in platinum-resistant ovarian cancer. Semin Cancer Biol. (2021) 77:42–55. doi: 10.1016/j.semcancer.2021.03.031 33812984

[58] Xu T Liu Z Huang L Jing J Liu X. Modulating the tumor immune microenvironment with nanoparticles: A sword for improving the efficiency of ovarian cancer immunotherapy. Front Immunol. (2022) 13:1057850. doi: 10.3389/fimmu.2022.1057850 36532066 PMC9751906

[59] Schweer D McAtee A Neupane K Richards C Ueland F Kolesar J. Tumor-associated macrophages and ovarian cancer: implications for therapy. Cancers (Basel). (2022) 14:2220. doi: 10.3390/cancers14092220 35565348 PMC9101750

[60] Fan Z Han D Fan X Zhao L. Ovarian cancer treatment and natural killer cell-based immunotherapy. Front Immunol. (2023) 14:1308143. doi: 10.3389/fimmu.2023.1308143 38187402 PMC10768003

[61] Blanc-Durand F Clemence Wei Xian L Tan DSP. Targeting the immune microenvironment for ovarian cancer therapy. Front Immunol. (2023) 14:1328651. doi: 10.3389/fimmu.2023.1328651 38164130 PMC10757966

[62] Li H Luo F Jiang X Zhang W Xiang T Pan Q. CircITGB6 promotes ovarian cancer cisplatin resistance by resetting tumor-associated macrophage polarization toward the M2 phenotype. J Immunother Cancer. (2022) 10:e004029. doi: 10.1136/jitc-2021-004029 35277458 PMC8919471

[63] Vilbois S Xu Y Ho PC. Metabolic interplay: tumor macrophages and regulatory T cells. Trends Cancer. (2024) 10:242–55. doi: 10.1016/j.trecan.2023.11.007 38135571

[64] Nasir I McGuinness C Poh AR Ernst M Darcy PK Britt KL. Tumor macrophage functional heterogeneity can inform the development of novel cancer therapies. Trends Immunol. (2023) 44:971–85. doi: 10.1016/j.it.2023.10.007 37995659

[65] Zeng W Li F Jin S Ho PC Liu PS Xie X. Functional polarization of tumor-associated macrophages dictated by metabolic reprogramming. J Exp Clin Cancer Res. (2023) 42:245. doi: 10.1186/s13046-023-02832-9 37740232 PMC10517486

[66] Liu J Cao X. Glucose metabolism of TAMs in tumor chemoresistance and metastasis. Trends Cell Biol. (2023) 33:967–78. doi: 10.1016/j.tcb.2023.03.008 37080816

[67] Lu CS Shiau AL Su BH Hsu TS Wang CT Su YC. Oct4 promotes M2 macrophage polarization through upregulation of macrophage colony-stimulating factor in lung cancer. J Hematol Oncol. (2020) 13:62. doi: 10.1186/s13045-020-00887-1 32487125 PMC7268452

[68] Parayath NN Gandham SK Leslie F Amiji MM. Improved anti-tumor efficacy of paclitaxel in combination with MicroRNA-125b-based tumor-associated macrophage repolarization in epithelial ovarian cancer. Cancer Lett. (2019) 461:1–9. doi: 10.1016/j.canlet.2019.07.002 31288064 PMC6682447

[69] Bai Y Yin K Su T Ji F Zhang S. CTHRC1 in ovarian cancer promotes M2-like polarization of tumor-associated macrophages via regulation of the STAT6 signaling pathway. OncoTargets Ther. (2020) 13:5743–53. doi: 10.2147/OTT.S250520 PMC7306458 32606786

[70] Xia H Li S Li X Wang W Bian Y Wei S. Autophagic adaptation to oxidative stress alters peritoneal residential macrophage survival and ovarian cancer metastasis. JCI Insight. (2020) 5:e141115. doi: 10.1172/jci.insight.141115 32780724 PMC7526547

[71] Hoover AA Hufnagel DH Harris W Bullock K Glass EB Liu E. Increased canonical NF-kappaB signaling specifically in macrophages is sufficient to limit tumor progression in syngeneic murine models of ovarian cancer. BMC Cancer. (2020) 20:970. doi: 10.1186/s12885-020-07450-8 33028251 PMC7542116

[72] Ardighieri L Missale F Bugatti M Gatta LB Pezzali I Monti M. Infiltration by CXCL10 secreting macrophages is associated with antitumor immunity and response to therapy in ovarian cancer subtypes. Front Immunol. (2021) 12:690201. doi: 10.3389/fimmu.2021.690201 34220848 PMC8253056

[73] Le F Yang L Han Y Zhong Y Zhan F Feng Y. TPL inhibits the invasion and migration of drug-resistant ovarian cancer by targeting the PI3K/AKT/NF-κB-signaling pathway to inhibit the polarization of M2 TAMs. Front Oncol. (2021) 11:704001. doi: 10.3389/fonc.2021.704001 34381726 PMC8350572

[74] Long L Hu Y Long T Lu X Tuo Y Li Y. Tumor-associated macrophages induced spheroid formation by CCL18-ZEB1-M-CSF feedback loop to promote transcoelomic metastasis of ovarian cancer. J Immunother Cancer. (2021) 9:e003973. doi: 10.1136/jitc-2021-003973 34969774 PMC8718465

[75] Khan ANH Emmons TR Magner WJ Alqassim E Singel KL Ricciuti J. VSSP abrogates murine ovarian tumor-associated myeloid cell-driven immune suppression and induces M1 polarization in tumor-associated macrophages from ovarian cancer patients. Cancer Immunol Immunother. (2022) 71:2355–69. doi: 10.1007/s00262-022-03156-x PMC10591410 35166871

[76] Asare-Werehene M Tsuyoshi H Zhang H Salehi R Chang CY Carmona E. Plasma gelsolin confers chemoresistance in ovarian cancer by resetting the relative abundance and function of macrophage subtypes. Cancers (Basel). (2022) 14:1039. doi: 10.3390/cancers14041039 35205790 PMC8870487

[77] Chen H Huang S Niu P Zhu Y Zhou J Jiang L. Cardamonin suppresses pro-tumor function of macrophages by decreasing M2 polarization on ovarian cancer cells via mTOR inhibition. Mol Ther Oncolytics. (2022) 26:175–88. doi: 10.1016/j.omto.2022.06.009 PMC9278033 35860007

[78] Yin L Wang Y. Extracellular vesicles derived from M2-polarized tumor-associated macrophages promote immune escape in ovarian cancer through NEAT1/miR-101–3p/ZEB1/PD-L1 axis. Cancer Immunol Immunother. (2023) 72:743–58. doi: 10.1007/s00262-022-03305-2 PMC10992138 36319716

[79] Wang Y He M Zhang C Cao K Zhang G Yang M. Siglec-9+ tumor-associated macrophages delineate an immunosuppressive subset with therapeutic vulnerability in patients with high-grade serous ovarian cancer. J Immunother Cancer. (2023) 11:e007099. doi: 10.1136/jitc-2023-007099 37709296 PMC10503378

[80] Brauneck F Oliveira-Ferrer L Muschhammer J Sturmheit T Ackermann C Haag F. Immunosuppressive M2 TAMs represent a promising target population to enhance phagocytosis of ovarian cancer cells in vitro. Front Immunol. (2023) 14:1250258. doi: 10.3389/fimmu.2023.1250258 37876933 PMC10593434

[81] Chhabra Y Weeraratna AT. Fibroblasts in cancer: Unity in heterogeneity. Cell. (2023) 186:1580–609. doi: 10.1016/j.cell.2023.03.016 PMC11422789 37059066

[82] Zhang X Zhang M Sun H Wang X Wang X Sheng W. The role of transcription factors in the crosstalk between cancer-associated fibroblasts and tumor cells. J advanced Res. (2024). doi: 10.1016/j.jare.2024.01.033 PMC11725164 38309692

[83] Kim MJ Jung D Park JY Lee SM An HJ. GLIS1 in cancer-associated fibroblasts regulates the migration and invasion of ovarian cancer cells. Int J Mol Sci. (2022) 23:2218. doi: 10.3390/ijms23042218 35216340 PMC8874490

[84] Akinjiyan FA Dave RM Alpert E Longmore GD Fuh KC. DDR2 expression in cancer-associated fibroblasts promotes ovarian cancer tumor invasion and metastasis through periostin-ITGB1. Cancers (Basel). (2022) 14:3482. doi: 10.3390/cancers14143482 35884543 PMC9319689

[85] Lin SC Liao YC Chen PM Yang YY Wang YH Tung SL. Periostin promotes ovarian cancer metastasis by enhancing M2 macrophages and cancer-associated fibroblasts via integrin-mediated NF-κB and TGF-β2 signaling. J BioMed Sci. (2022) 29:109. doi: 10.1186/s12929-022-00888-x 36550569 PMC9784270

[86] Akinjiyan FA Ibitoye Z Zhao P Shriver LP Patti GJ Longmore GD. DDR2-regulated arginase activity in ovarian cancer-associated fibroblasts promotes collagen production and tumor progression. Oncogene. (2024) 43:189–201. doi: 10.1038/s41388-023-02884-3 37996700 PMC10786713

[87] Guo H Ha C Dong H Yang Z Ma Y Ding Y. Cancer-associated fibroblast-derived exosomal microRNA-98–5p promotes cisplatin resistance in ovarian cancer by targeting CDKN1A. Cancer Cell Int. (2019) 19:347. doi: 10.1186/s12935-019-1051-3 31889899 PMC6925473

[88] Cui Y Wang D Xie M. Tumor-derived extracellular vesicles promote activation of carcinoma-associated fibroblasts and facilitate invasion and metastasis of ovarian cancer by carrying miR-630. Front Cell Dev Biol. (2021) 9:652322. doi: 10.3389/fcell.2021.652322 34277601 PMC8277948

[89] Sun L Ke M Wang X Yin M Wei J Xu L. FAP(high) α-SMA(low) cancer-associated fibroblast-derived SLPI protein encapsulated in extracellular vesicles promotes ovarian cancer development via activation of PI3K/AKT and downstream signaling pathways. Mol carcinogenesis. (2022) 61:910–23. doi: 10.1002/mc.23445 PMC9541539 35801406

[90] Mo Y Leung LL Mak CSL Wang X Chan WS Hui LMN. Tumor-secreted exosomal miR-141 activates tumor-stroma interactions and controls premetastatic niche formation in ovarian cancer metastasis. Mol Cancer. (2023) 22:4. doi: 10.1186/s12943-022-01703-9 36624516 PMC9827705

[91] Han Q Tan S Gong L Li G Wu Q Chen L. Omental cancer-associated fibroblast-derived exosomes with low microRNA-29c-3p promote ovarian cancer peritoneal metastasis. Cancer Sci. (2023) 114:1929–42. doi: 10.1111/cas.15726 PMC10154903 36644823

[92] Sun L Ke M Yin M Zeng Y Ji Y Hu Y. Extracellular vesicle-encapsulated microRNA-296–3p from cancer-associated fibroblasts promotes ovarian cancer development through regulation of the PTEN/AKT and SOCS6/STAT3 pathways. Cancer Sci. (2024) 115:155–69. doi: 10.1111/cas.16014 PMC10823290 37972389

[93] Liu J Liu C Ma Y Pan X Chu R Yao S. STING inhibitors sensitize platinum chemotherapy in ovarian cancer by inhibiting the CGAS-STING pathway in cancer-associated fibroblasts (CAFs). Cancer Lett. (2024) 588:216700. doi: 10.1016/j.canlet.2024.216700 38373690

[94] Jiang B Xiao S Zhang S Xiao F. The miR-1290/OGN axis in ovarian cancer-associated fibroblasts modulates cancer cell proliferation and invasion. J Ovarian Res. (2024) 17:52. doi: 10.1186/s13048-024-01364-w 38402185 PMC10893657

[95] Thongchot S Jamjuntra P Therasakvichya S Warnnissorn M Ferraresi A Thuwajit P. Interleukin−8 released by cancer−associated fibroblasts attenuates the autophagy and promotes the migration of ovarian cancer cells. Int J Oncol. (2021) 58:14. doi: 10.3892/ijo 33649784 PMC7949624

[96] Ji Z Tian W Gao W Zang R Wang H Yang G. Cancer-associated fibroblast-derived interleukin-8 promotes ovarian cancer cell stemness and Malignancy through the notch3-mediated signaling. Front Cell Dev Biol. (2021) 9:684505. doi: 10.3389/fcell.2021.684505 34277625 PMC8280773

[97] Jin Y Bian S Wang H Mo J Fei H Li L. CRMP2 derived from cancer associated fibroblasts facilitates progression of ovarian cancer via HIF-1α-glycolysis signaling pathway. Cell Death Dis. (2022) 13:675. doi: 10.1038/s41419-022-05129-5 35927239 PMC9352901

[98] Dai JM Sun K Li C Cheng M Guan JH Yang LN. Cancer-associated fibroblasts contribute to cancer metastasis and apoptosis resistance in human ovarian cancer via paracrine SDF-1α. Clin Transl Oncol. (2023) 25:1606–16. doi: 10.1007/s12094-022-03054-9 36593384

[99] Lasser SA Ozbay Kurt FG Arkhypov I Utikal J Umansky V. Myeloid-derived suppressor cells in cancer and cancer therapy. Nat Rev Clin Oncol. (2024) 21:147–64. doi: 10.1038/s41571-023-00846-y 38191922

[100] Wang J Ling D Shi L Li H Peng M Wen H. METTL3-mediated m6A methylation regulates ovarian cancer progression by recruiting myeloid-derived suppressor cells. Cell bioscience. (2023) 13:202. doi: 10.1186/s13578-023-01149-6 37932814 PMC10629157

[101] Ozbay Kurt FG Lasser S Arkhypov I Utikal J Umansky V. Enhancing immunotherapy response in melanoma: myeloid-derived suppressor cells as a therapeutic target. J Clin Invest. (2023) 133:e170762. doi: 10.1172/JCI170762 37395271 PMC10313369

[102] Barry ST Gabrilovich DI Sansom OJ Campbell AD Morton JP. Therapeutic targeting of tumour myeloid cells. Nat Rev Cancer. (2023) 23:216–37. doi: 10.1038/s41568-022-00546-2 36747021

[103] Arpinati L Scherz-Shouval R. From gatekeepers to providers: regulation of immune functions by cancer-associated fibroblasts. Trends Cancer. (2023) 9:421–43. doi: 10.1016/j.trecan.2023.01.007 36870916

[104] Youn JI Nagaraj S Collazo M Gabrilovich DI. Subsets of myeloid-derived suppressor cells in tumor-bearing mice. J Immunol. (2008) 181:5791–802. doi: 10.4049/jimmunol.181.8.5791 PMC2575748 18832739

[105] Wu L Deng Z Peng Y Han L Liu J Wang L. Ascites-derived IL-6 and IL-10 synergistically expand CD14+HLA-DR-/low myeloid-derived suppressor cells in ovarian cancer patients. Oncotarget. (2017) 8:76843–56. doi: 10.18632/oncotarget.v8i44 PMC5652747 29100353

[106] Okła K Czerwonka A Wawruszak A Bobiński M Bilska M Tarkowski R. Clinical relevance and immunosuppressive pattern of circulating and infiltrating subsets of myeloid-derived suppressor cells (MDSCs) in epithelial ovarian cancer. Front Immunol. (2019) 10:691. doi: 10.3389/fimmu.2019.00691 31001284 PMC6456713

[107] Horikawa N Abiko K Matsumura N Baba T Hamanishi J Yamaguchi K. Anti-VEGF therapy resistance in ovarian cancer is caused by GM-CSF-induced myeloid-derived suppressor cell recruitment. Br J Cancer. (2020) 122:778–88. doi: 10.1038/s41416-019-0725-x PMC7078258 31932754

[108] Li X Wang J Wu W Gao H Liu N Zhan G. Myeloid-derived suppressor cells promote epithelial ovarian cancer cell stemness by inducing the CSF2/p-STAT3 signalling pathway. FEBS J. (2020) 287:5218–35. doi: 10.1111/febs.15311 PMC7754107 32239647

[109] Okła K Rajtak A Czerwonka A Bobiński M Wawruszak A Tarkowski R. Accumulation of blood-circulating PD-L1-expressing M-MDSCs and monocytes/macrophages in pretreatment ovarian cancer patients is associated with soluble PD-L1. J Trans Med. (2020) 18:220. doi: 10.1186/s12967-020-02389-7 PMC7268341 32487171

[110] McGray AJR Eppolito C Miliotto A Singel KL Stephenson K Lugade A. A prime/boost vaccine platform efficiently identifies CD27 agonism and depletion of myeloid-derived suppressor cells as therapies that rationally combine with checkpoint blockade in ovarian cancer. Cancer Immunol Immunother. (2021) 70:3451–60. doi: 10.1007/s00262-021-02936-1 PMC8057655 33880648

[111] Yang Q Yu B Kang J Li A Sun J. Obesity promotes tumor immune evasion in ovarian cancer through increased production of myeloid-derived suppressor cells via IL-6. Cancer Manage Res. (2021) 13:7355–63. doi: 10.2147/CMAR.S303707 PMC8464309 34584460

[112] Chen H Yang K Pang L Fei J Zhu Y Zhou J. ANKRD22 is a potential novel target for reversing the immunosuppressive effects of PMN-MDSCs in ovarian cancer. J Immunother Cancer. (2023) 11:e005527. doi: 10.1136/jitc-2022-005527 36822671 PMC9950970

[113] Fang X Svitkina TM. Adenomatous Polyposis Coli (APC) in cell migration. Eur J Cell Biol. (2022) 101:151228. doi: 10.1016/j.ejcb.2022.151228 35483122 PMC9357102

[114] Huang Q Wang F Hao D Li X Li X Lei T. Deciphering tumor-infiltrating dendritic cells in the single-cell era. Exp Hematol Oncol. (2023) 12:97. doi: 10.1186/s40164-023-00459-2 38012715 PMC10680280

[115] Del Prete A Salvi V Soriani A Laffranchi M Sozio F Bosisio D. Dendritic cell subsets in cancer immunity and tumor antigen sensing. Cell Mol Immunol. (2023) 20:432–47. doi: 10.1038/s41423-023-00990-6 PMC10203372 36949244

[116] Zhang C Wang K Wang H. Adenosine in cancer immunotherapy: Taking off on a new plane. Biochim Biophys Acta Rev Cancer. (2023) 1878:189005. doi: 10.1016/j.bbcan.2023.189005 37913941

[117] Gao Y Xu Y Zhao S Qian L Song T Zheng J. Growth differentiation factor-15 promotes immune escape of ovarian cancer via targeting CD44 in dendritic cells. Exp Cell Res. (2021) 402:112522. doi: 10.1016/j.yexcr.2021.112522 33771482

[118] Luo Y Shreeder B Jenkins JW Shi H Lamichhane P Zhou K. Th17-inducing dendritic cell vaccines stimulate effective CD4 T cell-dependent antitumor immunity in ovarian cancer that overcomes resistance to immune checkpoint blockade. J Immunother Cancer. (2023) 11:e007661. doi: 10.1136/jitc-2023-007661 37918918 PMC10626769

[119] Zhang H Yang L Wang T Li Z. NK cell-based tumor immunotherapy. Bioactive materials. (2024) 31:63–86. doi: 10.1016/j.bioactmat.2023.08.001 37601277 PMC10432724

[120] Peng S Lin A Jiang A Zhang C Zhang J Cheng Q. CTLs heterogeneity and plasticity: implications for cancer immunotherapy. Mol Cancer. (2024) 23:58. doi: 10.1186/s12943-024-01972-6 38515134 PMC10956324

[121] Page A Chuvin N Valladeau-Guilemond J Depil S. Development of NK cell-based cancer immunotherapies through receptor engineering. Cell Mol Immunol. (2024) 21:315–31. doi: 10.1038/s41423-024-01145-x PMC10978891 38443448

[122] Borde S Matosevic S. Metabolic adaptation of NK cell activity and behavior in tumors: challenges and therapeutic opportunities. Trends Pharmacol Sci. (2023) 44:832–48. doi: 10.1016/j.tips.2023.08.009 37770314

[123] Wang K Wang L Wang Y Xiao L Wei J Hu Y. Reprogramming natural killer cells for cancer therapy. Mol Ther. (2024). doi: 10.1016/j.ymthe.2024.01.027 PMC11403237 38273655

[124] Van der Meer JMR Maas RJA Guldevall K Klarenaar K de Jonge P Evert JSH. IL-15 superagonist N-803 improves IFNγ production and killing of leukemia and ovarian cancer cells by CD34(+) progenitor-derived NK cells. Cancer Immunol Immunother. (2021) 70:1305–21. doi: 10.1007/s00262-020-02749-8 PMC8053152 33140189

[125] Van der Meer JMR de Jonge P van der Waart AB Geerlings AC Moonen JP Brummelman J. CD34(+) progenitor-derived NK cell and gemcitabine combination therapy increases killing of ovarian cancer cells in NOD/SCID/IL2Rg(null) mice. Oncoimmunology. (2021) 10:1981049. doi: 10.1080/2162402X.2021.1981049 34616589 PMC8489932

[126] van Vloten JP Matuszewska K Minow MAA Minott JA Santry LA Pereira M. Oncolytic Orf virus licenses NK cells via cDC1 to activate innate and adaptive antitumor mechanisms and extends survival in a murine model of late-stage ovarian cancer. J Immunother Cancer. (2022) 10:e004335. doi: 10.1136/jitc-2021-004335 35296558 PMC8928368

[127] Fraser CC Jia B Hu G Al Johani LI Fritz-Klaus R Ham JD. Ovarian cancer ascites inhibits transcriptional activation of NK cells partly through CA125. J Immunol. (2022) 208:2227–38. doi: 10.4049/jimmunol.2001095 PMC10852100 35396222

[128] Raja R Wu C Bassoy EY Rubino TE Jr. Utagawa EC Magtibay PM. PP4 inhibition sensitizes ovarian cancer to NK cell-mediated cytotoxicity via STAT1 activation and inflammatory signaling. J Immunother Cancer. (2022) 10:e005026. doi: 10.1101/2022.08.25.505192 36564125 PMC9791393

[129] Luo H Zhou Y Zhang J Zhang Y Long S Lin X. NK cell-derived exosomes enhance the anti-tumor effects against ovarian cancer by delivering cisplatin and reactivating NK cell functions. Front Immunol. (2022) 13:1087689. doi: 10.3389/fimmu.2022.1087689 36741396 PMC9892755

[130] Steitz AM Schröder C Knuth I Keber CU Sommerfeld L Finkernagel F. TRAIL-dependent apoptosis of peritoneal mesothelial cells by NK cells promotes ovarian cancer invasion. iScience. (2023) 26:108401. doi: 10.1016/j.isci.2023.108401 38047087 PMC10692662

[131] Osum KC Jenkins MK. Toward a general model of CD4(+) T cell subset specification and memory cell formation. Immunity. (2023) 56:475–84. doi: 10.1016/j.immuni.2023.02.010 PMC10084496 36921574

[132] Shah K Al-Haidari A Sun J Kazi JU. T cell receptor (TCR) signaling in health and disease. Signal Transduct Target Ther. (2021) 6:412. doi: 10.1038/s41392-021-00823-w 34897277 PMC8666445

[133] Klement JD Redd PS Lu C Merting AD Poschel DB Yang D. Tumor PD-L1 engages myeloid PD-1 to suppress type I interferon to impair cytotoxic T lymphocyte recruitment. Cancer Cell. (2023) 41:620–36.e9. doi: 10.1016/j.ccell.2023.02.005 36917954 PMC10150625

[134] Chen F Xu Y Chen Y Shan S. TIGIT enhances CD4(+) regulatory T-cell response and mediates immune suppression in a murine ovarian cancer model. Cancer Med. (2020) 9:3584–91. doi: 10.1002/cam4.2976 PMC7221438 32212317

[135] Silveira HS Lupi LA Romagnoli GG Kaneno R da Silva Nunes I Fávaro WJ. P-MAPA activates TLR2 and TLR4 signaling while its combination with IL-12 stimulates CD4+ and CD8+ effector T cells in ovarian cancer. Life Sci. (2020) 254:117786. doi: 10.1016/j.lfs.2020.117786 32433918

[136] Asare-Werehene M Communal L Carmona E Han Y Song YS Burger D. Plasma gelsolin inhibits CD8(+) T-cell function and regulates glutathione production to confer chemoresistance in ovarian cancer. Cancer Res. (2020) 80:3959–71. doi: 10.1158/0008-5472.CAN-20-0788 32641415

[137] Desbois M Udyavar AR Ryner L Kozlowski C Guan Y Dürrbaum M. Integrated digital pathology and transcriptome analysis identifies molecular mediators of T-cell exclusion in ovarian cancer. Nat Commun. (2020) 11:5583. doi: 10.1038/s41467-020-19408-2 33149148 PMC7642433

[138] McCaw TR Goel N Brooke DJ Katre AA Londoño AI Smith HJ. Class I histone deacetylase inhibition promotes CD8 T cell activation in ovarian cancer. Cancer Med. (2021) 10:709–17. doi: 10.1002/cam4.3337 PMC7877343 33369199

[139] Sima LE Chen S Cardenas H Zhao G Wang Y Ivan C. Loss of host tissue transglutaminase boosts antitumor T cell immunity by altering STAT1/STAT3 phosphorylation in ovarian cancer. J Immunother Cancer. (2021) 9:e002682. doi: 10.1136/jitc-2021-002682 34593619 PMC8487211

[140] Muthuswamy R McGray AR Battaglia S He W Miliotto A Eppolito C. CXCR6 by increasing retention of memory CD8(+) T cells in the ovarian tumor microenvironment promotes immunosurveillance and control of ovarian cancer. J Immunother Cancer. (2021) 9:e003329. doi: 10.1136/jitc-2021-003329 34607898 PMC8491420

[141] Tsuji T Matsuzaki J Odunsi K. Tissue residency of memory CD8(+) T cells matters in shaping immunogenicity of ovarian cancer. Cancer Cell. (2022) 40:452–4. doi: 10.1016/j.ccell.2022.04.008 PMC11778921 35537410

[142] Kamat K Krishnan V Dorigo O. Macrophage-derived CCL23 upregulates expression of T-cell exhaustion markers in ovarian cancer. Br J Cancer. (2022) 127:1026–33. doi: 10.1038/s41416-022-01887-3 PMC9470573 35750747

[143] Zhu XL Wang HJ Wang XR Wu D Ji X Xu L. IL-6 secretion of CD4(+) T cells stimulated by LC3-positive extracellular vesicles in human epithelial ovarian cancer. Clin Transl Oncol. (2022) 24:2222–30. doi: 10.1007/s12094-022-02883-y 35871126

[144] Zhang J He T Yin Z Shang C Xue L Guo H. Ascitic senescent T cells are linked to chemoresistance in patients with advanced high-grade serous ovarian cancer. Front Oncol. (2022) 12:864021. doi: 10.3389/fonc.2022.864021 35875098 PMC9301961

[145] Vlaming M Bilemjian V Freile J Melo V Plat A Huls G. Tumor infiltrating CD8/CD103/TIM-3-expressing lymphocytes in epithelial ovarian cancer co-express CXCL13 and associate with improved survival. Front Immunol. (2022) 13:1031746. doi: 10.3389/fimmu.2022.1031746 36341460 PMC9633842

[146] Yakubovich E Cook DP Rodriguez GM Vanderhyden BC. Mesenchymal ovarian cancer cells promote CD8(+) T cell exhaustion through the LGALS3-LAG3 axis. NPJ Syst Biol Appl. (2023) 9:61. doi: 10.1038/s41540-023-00322-4 38086828 PMC10716312

[147] Xu R Wu M Liu S Shang W Li R Xu J. Glucose metabolism characteristics and TLR8-mediated metabolic control of CD4(+) Treg cells in ovarian cancer cells microenvironment. Cell Death Dis. (2021) 12:22. doi: 10.1038/s41419-020-03272-5 33414414 PMC7790820

[148] Negura I Pavel-Tanasa M Danciu M. Regulatory T cells in gastric cancer: Key controllers from pathogenesis to therapy. Cancer Treat Rev. (2023) 120:102629. doi: 10.1016/j.ctrv.2023.102629 37769435

[149] Kang JH Zappasodi R. Modulating Treg stability to improve cancer immunotherapy. Trends Cancer. (2023) 9:911–27. doi: 10.1016/j.trecan.2023.07.015 PMC13390109 37598003

[150] Wang Y Huang T Gu J Lu L. Targeting the metabolism of tumor-infiltrating regulatory T cells. Trends Immunol. (2023) 44:598–612. doi: 10.1016/j.it.2023.06.001 37442660

[151] Shan Y Zhang B Chen L Zhang H Jiang C You Q. Herpesvirus entry mediator regulates the transduction of Tregs via STAT5/Foxp3 signaling pathway in ovarian cancer cells. Anti-cancer Drugs. (2023) 34:73–80. doi: 10.1097/CAD.0000000000001336 35946515

[152] Ippolito MR Martis V Martin S Tijhuis AE Hong C Wardenaar R. Gene copy-number changes and chromosomal instability induced by aneuploidy confer resistance to chemotherapy. Dev Cell. (2021) 56:2440–2454 e6. doi: 10.1016/j.devcel.2021.07.006 34352223

[153] Moore KN Bookman M Sehouli J Miller A Anderson C Scambia G. Atezolizumab, bevacizumab, and chemotherapy for newly diagnosed stage III or IV ovarian cancer: placebo-controlled randomized phase III trial (IMagyn050/GOG 3015/ENGOT-OV39). J Clin Oncol. (2021) 39:1842–55. doi: 10.1200/JCO.21.00306 PMC8189598 33891472

[154] Butti R Nimma R Kundu G Bulbule A Kumar TVS Gunasekaran VP. Tumor-derived osteopontin drives the resident fibroblast to myofibroblast differentiation through Twist1 to promote breast cancer progression. Oncogene. (2021) 40:2002–17. doi: 10.1038/s41388-021-01663-2 33603163

[155] Sadeghalvad M Mohammadi-Motlagh H-R Rezaei N. Immune microenvironment in different molecular subtypes of ductal breast carcinoma. Breast Cancer Res Treat. (2021) 185:261–79. doi: 10.1007/s10549-020-05954-2 33011829

[156] Salemme V Centonze G Cavallo F Defilippi P Conti L. The Crosstalk between Tumor Cells and the Immune Microenvironment in breast Cancer: implications for Immunotherapy. Front Oncol. (2021) 11:610303. doi: 10.3389/fonc.2021.610303 33777750 PMC7991834

[157] Flores-Balcazar CH Urias-Arce DM. Radiotherapy in women with epithelial ovarian cancer: historical role, current advances, and indications. Chin Clin Oncol. (2020) 9:49. doi: 10.21037/cco 32692187

[158] Kennedy LB Salama AKS. A review of cancer immunotherapy toxicity. CA Cancer J Clin. (2020) 70:86–104. doi: 10.3322/caac.21596 31944278

[159] Ratajczak K Grel H Olejnik P Jakiela S Stobiecka M. Current progress, strategy, and prospects of PD-1/PDL-1 immune checkpoint biosensing platforms for cancer diagnostics, therapy monitoring, and drug screening. Biosensors bioelectronics. (2023) 240:115644. doi: 10.1016/j.bios.2023.115644 37660460

[160] Yi M Zheng X Niu M Zhu S Ge H Wu K. Combination strategies with PD-1/PD-L1 blockade: current advances and future directions. Mol Cancer. (2022) 21:28. doi: 10.1186/s12943-021-01489-2 35062949 PMC8780712

[161] Sun Q Hong Z Zhang C Wang L Han Z Ma D. Immune checkpoint therapy for solid tumours: clinical dilemmas and future trends. Signal Transduct Target Ther. (2023) 8:320. doi: 10.1038/s41392-023-01522-4 37635168 PMC10460796

[162] Borgeaud M Sandoval J Obeid M Banna G Michielin O Addeo A. Novel targets for immune-checkpoint inhibition in cancer. Cancer Treat Rev. (2023) 120:102614. doi: 10.1016/j.ctrv.2023.102614 37603905

[163] Topalian SL Forde PM Emens LA Yarchoan M Smith KN Pardoll DM. Neoadjuvant immune checkpoint blockade: A window of opportunity to advance cancer immunotherapy. Cancer Cell. (2023) 41:1551–66. doi: 10.1016/j.ccell.2023.07.011 PMC10548441 37595586

[164] Guerra NL Matas-García A Serra-García L Morgado-Carrasco D Padrosa J Aldecoa I. Dermatomyositis unleashed by immune checkpoint inhibitors. Three additional cases and a review of the literature. Autoimmun Rev. (2023) 22:103375. doi: 10.1016/j.autrev.2023.103375 37321468 PMC10529928

[165] Wu M Huang Q Xie Y Wu X Ma H Zhang Y. Improvement of the anticancer efficacy of PD-1/PD-L1 blockade via combination therapy and PD-L1 regulation. J Hematol Oncol. (2022) 15:24. doi: 10.1186/s13045-022-01242-2 35279217 PMC8917703

[166] Fukumoto T Fatkhutdinov N Zundell JA Tcyganov EN Nacarelli T Karakashev S. HDAC6 inhibition synergizes with anti-PD-L1 therapy in ARID1A-inactivated ovarian cancer. Cancer Res. (2019) 79:5482–9. doi: 10.1158/0008-5472.CAN-19-1302 PMC6825538 31311810

[167] Shang A Wang W Gu C Chen C Zeng B Yang Y. Long non-coding RNA HOTTIP enhances IL-6 expression to potentiate immune escape of ovarian cancer cells by upregulating the expression of PD-L1 in neutrophils. J Exp Clin Cancer Res. (2019) 38:411. doi: 10.1186/s13046-019-1394-6 31533774 PMC6751824

[168] Cai D Li J Liu D Hong S Qiao Q Sun Q. Tumor-expressed B7-H3 mediates the inhibition of antitumor T-cell functions in ovarian cancer insensitive to PD-1 blockade therapy. Cell Mol Immunol. (2020) 17:227–36. doi: 10.1038/s41423-019-0305-2 PMC7051965 31611650

[169] Lampert EJ Zimmer A Padget M Cimino-Mathews A Nair JR Liu Y. Combination of PARP inhibitor olaparib, and PD-L1 inhibitor durvalumab, in recurrent ovarian cancer: a proof-of-concept phase II study. Clin Cancer Res. (2020) 26:4268–79. doi: 10.1158/1078-0432.CCR-20-0056 PMC7442720 32398324

[170] Zhang QF Li J Jiang K Wang R Ge JL Yang H. CDK4/6 inhibition promotes immune infiltration in ovarian cancer and synergizes with PD-1 blockade in a B cell-dependent manner. Theranostics. (2020) 10:10619–33. doi: 10.7150/thno.44871 PMC7482823 32929370

[171] Wan C Keany MP Dong H Al-Alem LF Pandya UM Lazo S. Enhanced efficacy of simultaneous PD-1 and PD-L1 immune checkpoint blockade in high-grade serous ovarian cancer. Cancer Res. (2021) 81:158–73. doi: 10.1158/0008-5472.CAN-20-1674 PMC7878408 33158814

[172] Yang M Lu J Zhang G Wang Y He M Xu Q. CXCL13 shapes immunoactive tumor microenvironment and enhances the efficacy of PD-1 checkpoint blockade in high-grade serous ovarian cancer. J Immunother Cancer. (2021) 9:e001136. doi: 10.1136/jitc-2020-001136 33452206 PMC7813306

[173] Laumont CM Wouters MCA Smazynski J Gierc NS Chavez EA Chong LC. Single-cell profiles and prognostic impact of tumor-infiltrating lymphocytes coexpressing CD39, CD103, and PD-1 in ovarian cancer. Clin Cancer Res. (2021) 27:4089–100. doi: 10.1158/1078-0432.CCR-20-4394 33963000

[174] Seitz S Dreyer TF Stange C Steiger K Bräuer R Scheutz L. CXCL9 inhibits tumour growth and drives anti-PD-L1 therapy in ovarian cancer. Br J Cancer. (2022) 126:1470–80. doi: 10.1038/s41416-022-01763-0 PMC9090786 35314795

[175] Dong M Qian M Ruan Z. CUL3/SPOP complex prevents immune escape and enhances chemotherapy sensitivity of ovarian cancer cells through degradation of PD-L1 protein. J Immunother Cancer. (2022) 10:e005270. doi: 10.1136/jitc-2022-005270 36198437 PMC9535172

[176] Wang Y Han J Wang D Cai M Xu Y Hu Y. Anti-PD-1 antibody armored γδ T cells enhance anti-tumor efficacy in ovarian cancer. Signal Transduct Target Ther. (2023) 8:399. doi: 10.1038/s41392-023-01646-7 37857598 PMC10587135

[177] Lo BC Kryczek I Yu J Vatan L Caruso R Matsumoto M. Microbiota-dependent activation of CD4(+) T cells induces CTLA-4 blockade-associated colitis via Fcgamma receptors. Science. (2024) 383:62–70. doi: 10.1126/science.adh8342 38175892 PMC12091338

[178] Wang SJ Dougan SK Dougan M. Immune mechanisms of toxicity from checkpoint inhibitors. Trends Cancer. (2023) 9:543–53. doi: 10.1016/j.trecan.2023.04.002 PMC10330206 37117135

[179] Pulanco MC Madsen AT Tanwar A Corrigan DT Zang X. Recent advancements in the B7/CD28 immune checkpoint families: new biology and clinical therapeutic strategies. Cell Mol Immunol. (2023) 20:694–713. doi: 10.1038/s41423-023-01019-8 37069229 PMC10310771

[180] Sharma P Goswami S Raychaudhuri D Siddiqui BA Singh P Nagarajan A. Immune checkpoint therapy-current perspectives and future directions. Cell. (2023) 186:1652–69. doi: 10.1016/j.cell.2023.03.006 37059068

[181] Friese C Harbst K Borch TH Westergaard MCW Pedersen M Kverneland A. CTLA-4 blockade boosts the expansion of tumor-reactive CD8(+) tumor-infiltrating lymphocytes in ovarian cancer. Sci Rep. (2020) 10:3914. doi: 10.1038/s41598-020-60738-4 32127601 PMC7054305

[182] Świderska J Kozłowski M Nowak K Rychlicka M Branecka-Woźniak D Kwiatkowski S. Clinical Relevance of Soluble Forms of Immune Checkpoint Molecules sPD-1, sPD-L1, and sCTLA-4 in the Diagnosis and Prognosis of Ovarian Cancer. Diagnostics (Basel Switzerland). (2022) 12:189. doi: 10.3390/diagnostics12010189 35054356 PMC8774466

[183] Chen J Kang S Wu J Zhao J Si W Sun H. CTLA-4 polymorphism contributes to the genetic susceptibility of epithelial ovarian cancer. J obstetrics gynaecology Res. (2022) 48:1240–7. doi: 10.1111/jog.15186 35150042

[184] Peng M Mo Y Wang Y Wu P Zhang Y Xiong F. Neoantigen vaccine: an emerging tumor immunotherapy. Mol Cancer. (2019) 18:128. doi: 10.1186/s12943-019-1055-6 31443694 PMC6708248

[185] Melssen MM Sheybani ND Leick KM Slingluff CL Jr. Barriers to immune cell infiltration in tumors. J Immunother Cancer. (2023) 11:e006401. doi: 10.1136/jitc-2022-006401 37072352 PMC10124321

[186] Adams SF Grimm AJ Chiang CL Mookerjee A Flies D Jean S. Rapid tumor vaccine using Toll-like receptor-activated ovarian cancer ascites monocytes. J Immunother Cancer. (2020) 8:e000875. doi: 10.1136/jitc-2020-000875 32817208 PMC7430560

[187] Block MS Dietz AB Gustafson MP Kalli KR Erskine CL Youssef B. Th17-inducing autologous dendritic cell vaccination promotes antigen-specific cellular and humoral immunity in ovarian cancer patients. Nat Commun. (2020) 11:5173. doi: 10.1038/s41467-020-18962-z 33057068 PMC7560895

[188] Tanyi JL Chiang CL Chiffelle J Thierry AC Baumgartener P Huber F. Personalized cancer vaccine strategy elicits polyfunctional T cells and demonstrates clinical benefits in ovarian cancer. NPJ Vaccines. (2021) 6:36. doi: 10.1038/s41541-021-00332-5 33723260 PMC7960755

[189] Nishida S Morimoto S Oji Y Morita S Shirakata T Enomoto T. Cellular and humoral immune responses induced by an HLA class I-restricted peptide cancer vaccine targeting WT1 are associated with favorable clinical outcomes in advanced ovarian cancer. J immunotherapy (Hagerstown Md.: 1997). (2022) 45:56–66. doi: 10.1097/CJI.0000000000000405 PMC8654282 34874330

[190] Zhang L Zhao W Huang J Li F Sheng J Song H. Development of a dendritic cell/tumor cell fusion cell membrane nano-vaccine for the treatment of ovarian cancer. Front Immunol. (2022) 13:828263. doi: 10.3389/fimmu.2022.828263 35251013 PMC8893350

[191] Fucikova J Hensler M Kasikova L Lanickova T Pasulka J Rakova J. An autologous dendritic cell vaccine promotes anticancer immunity in patients with ovarian cancer with low mutational burden and cold tumors. Clin Cancer Res. (2022) 28:3053–65. doi: 10.1158/1078-0432.CCR-21-4413 35536547

[192] Zhao Z Ortega-Rivera OA Chung YH Simms A Steinmetz NF. A co-formulated vaccine of irradiated cancer cells and cowpea mosaic virus improves ovarian cancer rejection. J materials Chem B. (2023) 11:5429–41. doi: 10.1039/D2TB02355E PMC10293063 36861401

[193] Kos FJ Frankel P Cristea M Eng M Tinsley R Dempsey S. Immunologic signatures of peripheral blood T cells reveal the outcome of p53MVA vaccine and pembrolizumab treatment in patients with advanced ovarian cancer. Cancer Res Commun. (2023) 3:2585–95. doi: 10.1158/2767-9764.CRC-23-0394 PMC10732002 38032111

[194] Sterner RC Sterner RM. CAR-T cell therapy: current limitations and potential strategies. Blood Cancer J. (2021) 11:69. doi: 10.1038/s41408-021-00459-7 33824268 PMC8024391

[195] PI3Kdelta-CK1epsilon inhibitor umbralisib shows promise in R/R indolent NHL. Cancer Discovery. (2021) 11:1005. doi: 10.1158/2159-8290.CD-RW2021-040 33741708

[196] Li T Wang J. Therapeutic effect of dual CAR-T targeting PDL1 and MUC16 antigens on ovarian cancer cells in mice. BMC Cancer. (2020) 20:678. doi: 10.1186/s12885-020-07180-x 32689954 PMC7372885

[197] Shu R Evtimov VJ Hammett MV Nguyen NN Zhuang J Hudson PJ. Engineered CAR-T cells targeting TAG-72 and CD47 in ovarian cancer. Mol Ther Oncolytics. (2021) 20:325–41. doi: 10.1016/j.omto.2021.01.002 PMC7868933 33614914

[198] Schoutrop E El-Serafi I Poiret T Zhao Y Gultekin O He R. Mesothelin-specific CAR T cells target ovarian cancer. Cancer Res. (2021) 81:3022–35. doi: 10.1158/0008-5472.CAN-20-2701 33795251

[199] Guo C Dong E Lai Q Zhou S Zhang G Wu M. Effective antitumor activity of 5T4-specific CAR-T cells against ovarian cancer cells in *vitro* and xenotransplanted tumors in vivo. MedComm. (2020) 1:338–50. doi: 10.1002/mco2.34 PMC8491242 34766126

[200] Liang Z Dong J Yang N Li SD Yang ZY Huang R. Tandem CAR-T cells targeting FOLR1 and MSLN enhance the antitumor effects in ovarian cancer. Int J Biol Sci. (2021) 17:4365–76. doi: 10.7150/ijbs.63181 PMC8579462 34803504

[201] Chen J Hu J Gu L Ji F Zhang F Zhang M. Anti-mesothelin CAR-T immunotherapy in patients with ovarian cancer. Cancer Immunol Immunother. (2023) 72:409–25. doi: 10.1007/s00262-022-03238-w PMC10991348 35925286

[202] Shen Y Liu G Zhang Q Tian X Ouyang L Zhang L. Construction of CAR-T cells targeting TM4SF1 and its anti-tumor capacity in ovarian cancer. Immunol Lett. (2023) 255:1–9. doi: 10.1016/j.imlet.2023.01.011 36739093

[203] Schoutrop E Poiret T El-Serafi I Zhao Y He R Moter A. Tuned activation of MSLN-CAR T cells induces superior antitumor responses in ovarian cancer models. J Immunother Cancer. (2023) 11:e005691. doi: 10.1136/jitc-2022-005691 36746513 PMC9906404

[204] Ranoa DRE Sharma P Schane CP Lewis AN Valdez E Marada V. Single CAR-T cell treatment controls disseminated ovarian cancer in a syngeneic mouse model. J Immunother Cancer. (2023) 11:e006509. doi: 10.1136/jitc-2022-006509 37258040 PMC10255004

[205] Xu T Wang C Wang X Wang E Wang B Sun M. A novel TREM1/DAP12-based multiple chain CAR-T cell targets PTK7 in ovarian cancer therapy. Med Oncol (Northwood London England). (2023) 40:226. doi: 10.1007/s12032-023-02084-9 37405498

[206] Mun SS Meyerberg J Peraro L Korontsvit T Gardner T Malviya M. Dual targeting ovarian cancer by Muc16 CAR T cells secreting a bispecific T cell engager antibody for an intracellular tumor antigen WT1. Cancer Immunol Immunother. (2023) 72:3773–86. doi: 10.1007/s00262-023-03529-w PMC10991175 37635172

[207] Mondal T Gaur H Wamba BEN Michalak AG Stout C Watson MR. Characterizing the regulatory Fas (CD95) epitope critical for agonist antibody targeting and CAR-T bystander function in ovarian cancer. Cell Death Differ. (2023) 30:2408–31. doi: 10.1038/s41418-023-01229-7 PMC10657439 37838774

[208] Daigre J Martinez-Osuna M Bethke M Steiner L Dittmer V Krischer K. Preclinical evaluation of novel folate receptor 1-directed CAR T cells for ovarian cancer. Cancers (Basel). (2024) 16:333. doi: 10.3390/cancers16020333 38254822 PMC10813853

[209] Shalhout SZ Miller DM Emerick KS Kaufman HL. Therapy with oncolytic viruses: progress and challenges. Nat Rev Clin Oncol. (2023) 20:160–77. doi: 10.1038/s41571-022-00719-w 36631681

[210] Verhaak RG Tamayo P Yang JY Hubbard D Zhang H Creighton CJ. Prognostically relevant gene signatures of high-grade serous ovarian carcinoma. J Clin Investig. (2013) 123:517–25. doi: 10.1172/JCI65833 PMC3533304 23257362

[211] Zhang H Liu T Zhang Z Payne SH Zhang B McDermott JE. Integrated proteogenomic characterization of human high-grade serous ovarian cancer. Cell. (2016) 166:755–65. doi: 10.1016/j.cell.2016.05.069 PMC4967013 27372738

[212] Pearce OMT Delaine-Smith RM Maniati E Nichols S Wang J Böhm S. Deconstruction of a metastatic tumor microenvironment reveals a common matrix response in human cancers. Cancer Discovery. (2018) 8:304–19. doi: 10.1158/2159-8290.CD-17-0284 PMC5837004 29196464

[213] Dhawan U Wang SM Chu YH Huang GS Lin YR Hung YC. Nanochips of tantalum oxide nanodots as artificial-microenvironments for monitoring ovarian cancer progressiveness. Sci Rep. (2016) 6:31998. doi: 10.1038/srep31998 27534915 PMC4989222

[214] Bu X Mahoney KM Freeman GJ. Learning from PD-1 resistance: new combination strategies. Trends Mol Med. (2016) 22:448–51. doi: 10.1016/j.molmed.2016.04.008 PMC6833952 27174038

[215] Chester C Dorigo O Berek JS Kohrt H. Immunotherapeutic approaches to ovarian cancer treatment. J Immunother Cancer. (2015) 3:1–10. doi: 10.1186/s40425-015-0051-7 25806106 PMC4372273

[216] Tewari D Java JJ Salani R Armstrong DK Markman M Herzog T. Long-term survival advantage and prognostic factors associated with intraperitoneal chemotherapy treatment in advanced ovarian cancer: A gynecologic oncology group study. J Clin Oncol. (2015) 33:1460–6. doi: 10.1200/JCO.2014.55.9898 PMC4404424 25800756

[217] Owens GL Sheard VE Kalaitsidou M Blount D Lad Y Cheadle EJ. Preclinical assessment of CAR T-cell therapy targeting the tumor antigen 5T4 in ovarian cancer. J Immunother. (2018) 41:130–40. doi: 10.1097/CJI.0000000000000203 PMC5895166 29239915

